# Automated Personalized Feedback for Physical Activity and Dietary Behavior Change With Mobile Phones: A Randomized Controlled Trial on Adults

**DOI:** 10.2196/mhealth.4160

**Published:** 2015-05-14

**Authors:** Mashfiqui Rabbi, Angela Pfammatter, Mi Zhang, Bonnie Spring, Tanzeem Choudhury

**Affiliations:** ^1^ Cornell University Department of Information Science Ithaca, NY United States; ^2^ Northwestern University Department of Preventive Medicine Chicago, IL United States; ^3^ Michigan State University Department of Electrical and Computer Engineering East Lansing, MI United States

**Keywords:** mobile health, mHealth, mobile phone sensing, smart systems, context-aware systems, physical activity, self-management, personal health care, machine learning, artificial intelligence

## Abstract

**Background:**

A dramatic rise in health-tracking apps for mobile phones has occurred recently. Rich user interfaces make manual logging of users’ behaviors easier and more pleasant, and sensors make tracking effortless. To date, however, feedback technologies have been limited to providing overall statistics, attractive visualization of tracked data, or simple tailoring based on age, gender, and overall calorie or activity information. There are a lack of systems that can perform automated translation of behavioral data into specific actionable suggestions that promote healthier lifestyle without any human involvement.

**Objective:**

MyBehavior, a mobile phone app, was designed to process tracked physical activity and eating behavior data in order to provide personalized, actionable, low-effort suggestions that are contextualized to the user’s environment and previous behavior. This study investigated the technical feasibility of implementing an automated feedback system, the impact of the suggestions on user physical activity and eating behavior, and user perceptions of the automatically generated suggestions.

**Methods:**

MyBehavior was designed to (1) use a combination of automatic and manual logging to track physical activity (eg, walking, running, gym), user location, and food, (2) automatically analyze activity and food logs to identify frequent and nonfrequent behaviors, and (3) use a standard machine-learning, decision-making algorithm, called multi-armed bandit (MAB), to generate personalized suggestions that ask users to either continue, avoid, or make small changes to existing behaviors to help users reach behavioral goals. We enrolled 17 participants, all motivated to self-monitor and improve their fitness, in a pilot study of MyBehavior. In a randomized two-group trial, investigators randomly assigned participants to receive either MyBehavior’s personalized suggestions (n=9) or nonpersonalized suggestions (n=8), created by professionals, from a mobile phone app over 3 weeks. Daily activity level and dietary intake was monitored from logged data. At the end of the study, an in-person survey was conducted that asked users to subjectively rate their intention to follow MyBehavior suggestions.

**Results:**

In qualitative daily diary, interview, and survey data, users reported MyBehavior suggestions to be highly actionable and stated that they intended to follow the suggestions. MyBehavior users walked significantly more than the control group over the 3 weeks of the study (*P*=.05). Although some MyBehavior users chose lower-calorie foods, the between-group difference was not significant (*P*=.15). In a poststudy survey, users rated MyBehavior’s personalized suggestions more positively than the nonpersonalized, generic suggestions created by professionals (*P*<.001).

**Conclusions:**

MyBehavior is a simple-to-use mobile phone app with preliminary evidence of efficacy. To the best of our knowledge, MyBehavior represents the first attempt to create personalized, contextualized, actionable suggestions automatically from self-tracked information (ie, manual food logging and automatic tracking of activity). Lessons learned about the difficulty of manual logging and usability concerns, as well as future directions, are discussed.

**Trial Registration:**

ClinicalTrials.gov NCT02359981; https://clinicaltrials.gov/ct2/show/NCT02359981 (Archived by WebCite at http://www.webcitation.org/6YCeoN8nv).

## Introduction

In 2010, the World Health Organization (WHO) attributed 63% of deaths to noncommunicable diseases that are largely preventable [1]. The Centers for Disease Control and Prevention (CDC) estimates that in the US nearly 200,000 deaths annually could be prevented based upon modifications in diet, exercise, and obesity [2]. Obesity alone affects more than one-third of the adult population [3] and burdens the US with an estimated US $190 billion annually in health care costs [4].

A rapid rise has occurred in the development of mobile phone apps and wearable devices to address diet and physical activity. While empirical data is lacking for some commercial apps and sensor-based technologies [5,6], a number of scientific studies have explored the impact of novel technology-supported behavior change strategies on physical activity [7-9]. For example, Weegen et al [10] applied behavior change theories to design a mobile app that visualized a summary of physical activity logs and gave clinicians feedback to support their promotion of physical activity. Food logging has proved to be more difficult, burdensome, and time consuming than tracking physical activity. Recent work, however, has attempted to use image-based systems to decrease burden and enhance accuracy in food tracking with some success [11-13].  The ubiquity and ever-presence of mobile phones gives them the potential to perform assessment and intervention in the right place at the right time.

Although these methods show promise, they continue to fall short by not providing context-specific, relevant, personalized help at the moment when the individual needs it to make healthier choices. The science of how to present daily physical activity and dietary intake data back to users also has been at a suboptimal state.  To date, feedback has been limited to one of three categories: (1) overall numeric summaries [7,8,14] (eg, step counts), (2) tailored suggestions that only adapt to personal characteristics (eg, age, gender) and overall behavior (eg, daily calories consumed and burned) [15], and (3) visualizations that incorporate little processing [16].  Simple goals are offered, but without actionable insights on when, where, and how to achieve them. Visualization of large amounts of minimally processed data produces a related problem—information overload without clear steps to behavior change.  Providing personalized, in-the-moment, actionable guidance that prompts smaller, but more frequent, changes in existing behavior has potential for greater impact. A deeper look into physical activity and dietary intake data can reveal patterns of both healthy and unhealthy behavior that could be leveraged for personalized feedback. With current technologies, this can be achieved automatically, without human interpretation.

Given these observations, MyBehavior was created to address some shortcomings of current mHealth interventions. MyBehavior uses a machine-learning model—multi-armed bandit (MAB)—to automatically create contextualized and personalized suggestions based on the individual’s physical activity and dietary intake data collected solely from a mobile phone. Moreover, MyBehavior is one of the very few mHealth apps designed on the basis of established behavioral theory.  As such, the system reflects and incorporates the contemporary state of the behavioral science knowledge about how to foster healthful change.  Based on effective behavior change principles, MyBehavior provides low-effort suggestions that request small changes to users’ existing repeated behaviors.  To the best of our knowledge, MyBehavior is the first mHealth app that encourages healthy behavior change by automatically providing low-effort suggestions based on the user’s context and personal information.  

The objective of this study was to evaluate a new behavior change technology—MyBehavior—using a mixed-method approach as suggested by others [17]. We focused on (1) whether the users intended to follow the automated MyBehavior suggestions, (2) early indications of behavior change empowered by automated suggestions, and (3) participant feedback that could inform user experience and guide future design of automated health feedback systems.

The outcomes from this study will be used to further refine the features and messages of MyBehavior to optimize its effect on physical activity and dietary intake.

## Methods

### Study Procedure

To evaluate the feasibility of MyBehavior, a small 3-week, two-group randomized control trial (RCT) was conducted. The team that supervised the trial included the builders of the MyBehavior app and authors of this paper. This team recruited participants through advertisements placed around the Cornell University campus. In the advertisement, we invited participants to test a new mobile app to help them stay on track for physical activity and food intake. Recruitment was restricted to participants who owned an Android mobile phone and had an interest in fitness. Prior to the study, the investigators arranged face-to-face meetings with the participants and acquired their informed consent. Participants also completed a brief survey to provide demographic data and information about their prior experience with mobile technologies and weight loss/fitness apps. All participants attended a training session, where they installed MyBehavior on their primary mobile phone and received basic instructions, including how to enter their gender, height, and weight and how to set up a weekly weight goal (ie, lose weight, maintain weight, or gain weight). During the first week, users received a daily summary of their activities and food intake. This baseline week was intended to resemble many modern mobile health apps [5,6] without suggestions on what behaviors to change.

After the first week, the experimenters conducted an in-depth, semistructured interview with participants about their experience to date and then randomized participants into control and experimental groups. A random number generator was used for randomization. Assignment was single blind, as the study participants did not know their condition, while experimenters had full knowledge about the assignments.

We provided MyBehavior’s personalized context-sensitive suggestions to the experimental group, while the control group received generic prescriptive recommendations generated from a pool of 42 suggestions for healthy living, such as “walk for 30 minutes” and “eat fish for dinner.” A certified fitness professional created these generic suggestions after following National Institutes of Health resources [18,19].  An external nutrition counselor also reviewed the suggestions to ensure that they were both healthy and achievable. The list of these 42 suggestions is included as Multimedia Appendix 1 in this paper. For the following 2 weeks, participants continued to log behaviors and receive their respective suggestions on their mobile phones. During the entire study period, we asked participants to complete Web-based daily diaries to better understand their experience in following the suggestions provided. At the conclusion of the 3-week period, all participants were asked to complete a brief survey about the suggestions provided and were interviewed again face-to-face about their experience with the app.

This study was approved by Cornell University Institutional Review Board (1302003617) and a protocol was registered retrospectively at ClinicalTrails.gov (NCT02359981).

### Participants

**Figure 1:**
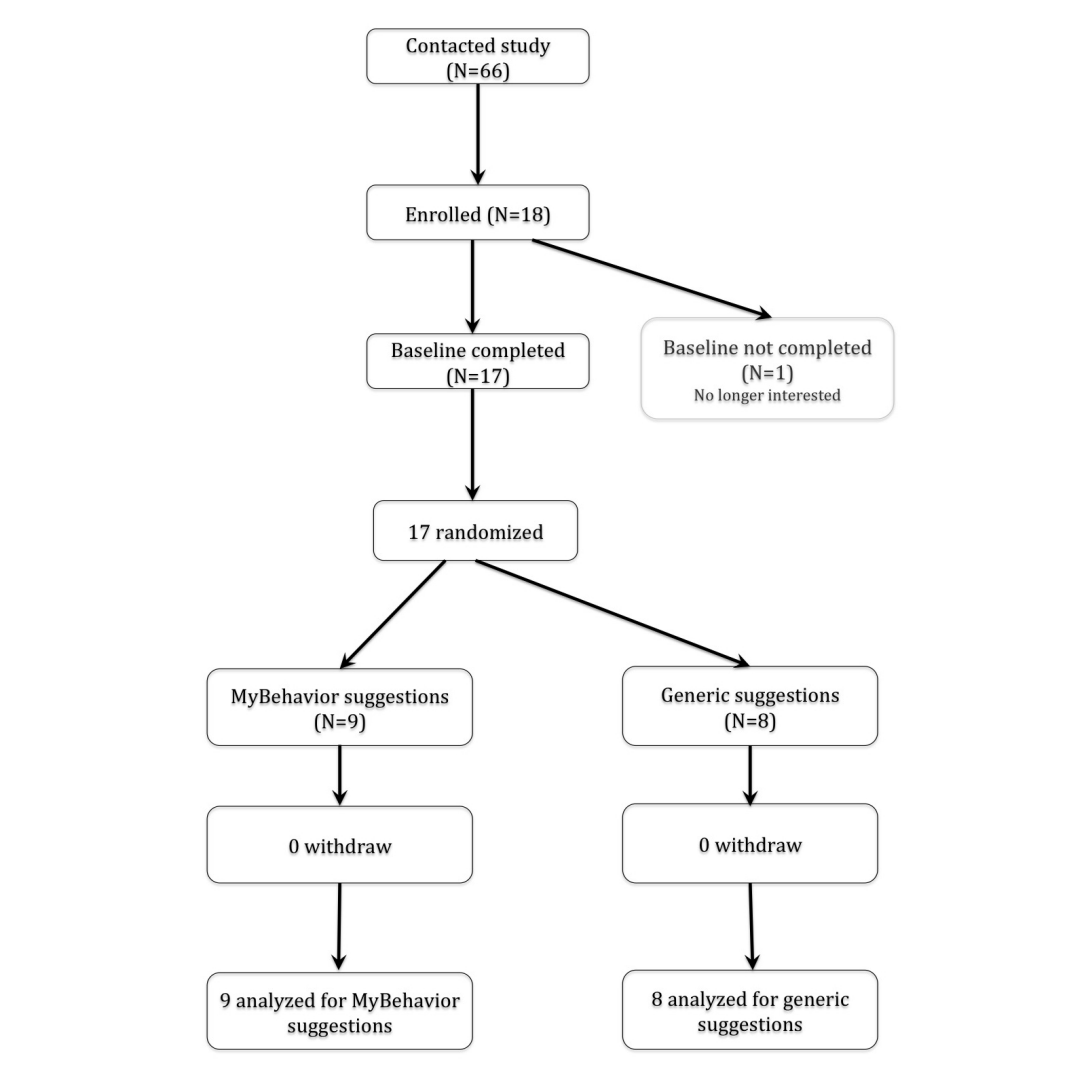
Flow of participants in the MyBehavior trial.

We recruited 18 participants, 17 of whom completed the study. Of the 17 participants, there were 13 students (76%), 4 professionals (24%), 8 females (47%), and 9 males (53%), and all were between the ages of 18 and 49 (mean 28.3, SD 6.96, lower quartile [q_25_]=22, median [q_50_]=26.3, upper quartile [q_75_]=36). All participants reported low-to-moderate levels of physical activity.  The majority of participants were experienced mobile phone users—9 participants (53%) had previous experience using a food diary, and 6 participants (35%) had previously kept an exercise log. After the randomization, participants in the groups were similar in terms of level of active lifestyle and experience with using mobile-based self-management tools. Our sample size was determined based on earlier literature [17,20,21] that suggested that small studies (n≥4) are more suitable to test early feasibility of novel behavior change technologies like MyBehavior. See Figure 1 for the flow of participants in the trial.

### MyBehavior Mobile App

#### Overview

MyBehavior is comprised of five key modules: (1) physical activity tracking, (2) food logging, (3) life-log generation, (4) physical activity and food clustering, and (5) suggestion generation.

#### Physical Activity Tracking

MyBehavior uses the accelerometer and the Global Positioning System (GPS) sensor inside the mobile phone to continuously keep track of an individual’s physical activities. A number of statistical features (eg, mean, variance, zero-crossing rate) are extracted from the sensor data and a machine-learning model—Gaussian Mixture Model (GMM) [22]—is applied to map the extracted feature values into the four most common daily physical activities—walking, running, stationary (sitting or standing), and driving. The technical details of this mobile sensing framework can be found in our previous work [23]. For physical activities that cannot be automatically recognized, MyBehavior provides users with a list of about 800 different physical activities from the compendium of physical activities [24]. Users can manually select the specific physical activity from the list and record the start and end time for the activity. In addition to tracking physical activities, MyBehavior calculates the calories expended during these activities based on the standard Metabolic Equivalents of Task (METS) [25].

#### Food Logging

Users select food items from a database and enter the consumed quantity to get the corresponding calorie intake. The United States Department of Agriculture (USDA) [26] maintains this database, containing more than 8000 types of food.

**Figure 2:**
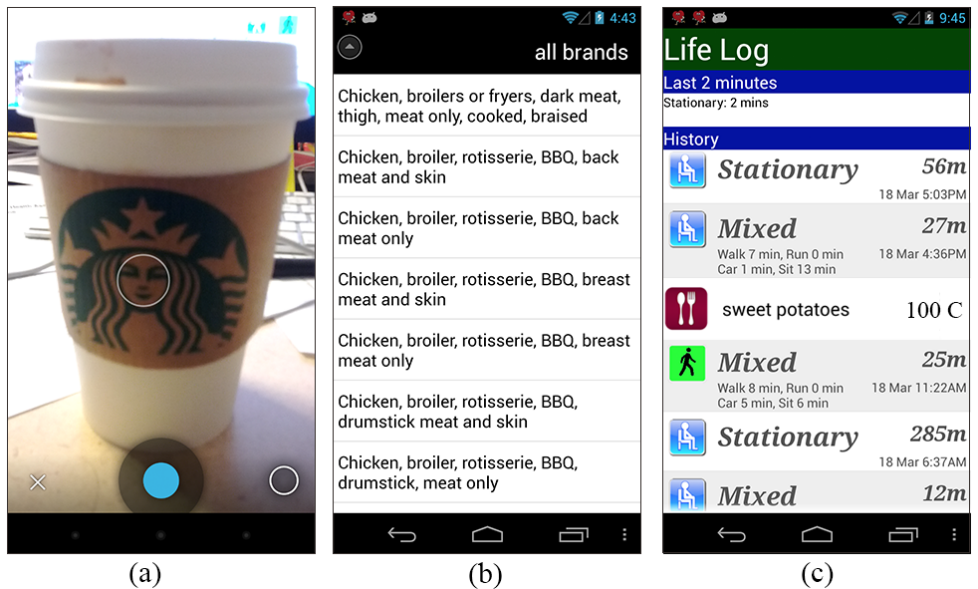
MyBehavior app screenshots: (a) taking photo of a food item, (b) searching for foods from the USDA database, and (c) Life Log, a chronological list of activity and food log events.

MyBehavior provides several features to make the food logging experience easier for users. First, users can take photos of their food. These photos serve as a memory aid when users are prompted to input their food information at 9:30pm every night (see Figure 2). Second, to facilitate entry of frequently repeated food choices, MyBehavior allows users to input food which reuses a prior meal (eg, add yesterday’s breakfast) and prioritizes food items that were selected previously. Finally, MyBehavior provides users with an option to directly input calorie information taken from the label of prepackaged foods (eg, a soft drink can or yogurt cup).

#### Life-Log Generation

MyBehavior generates as a “life log,” a chronological list of activity and food log events, as shown in Figure 2.  The log includes food, automatically sensed physical activity, and manually logged exercise entries as life events. To create concise and meaningful activity entries, MyBehavior processes the data into two stages.

In the first stage, activity predictions, which happen every 1 second, are aggregated every minute and labeled automatically. In the second stage, the contiguous activities having the same label are combined into a single entry. For example, if a user is stationary for 50 minutes, MyBehavior will generate a “stationary” activity entry into the life log with a 50-minute duration. Other common life events include a sequence of different activities that happen within a short time interval (ie, 15 minutes). An example might be the following:  walk to the bus stop, wait for a few minutes, ride the bus, and walk to the office after exiting the bus.  In this example, MyBehavior generates a “mixed” activity entry in the life log (eg, taking the bus from home to work) by combining the multiple activity sequences that happen within a 15-minute window.  

#### Physical Activity and Food Clustering

To enable suggestion generation delivery to the experimental group only, MyBehavior used the life logs to cluster similar physical activities and similar food items.  The food similarity matching process follows a simple logic—food is clustered based on similar food ingredients. For example, MyBehavior will detect if a user is repeatedly having high-calorie burgers with similar ingredients and form the cluster “burger” that groups together the same or similar types of burgers.  

**Figure 3:**
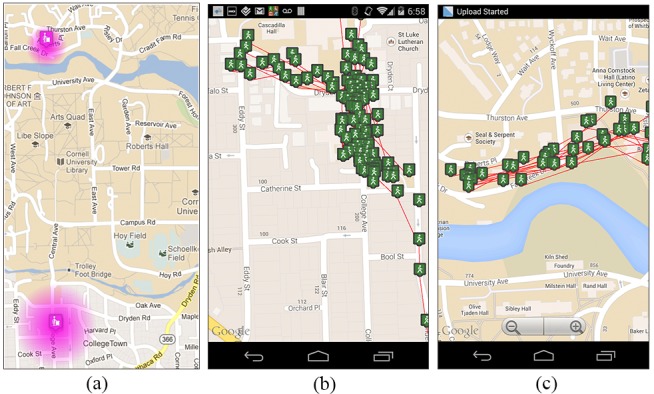
Clusters generated from user activities: (a) locations where user A stayed stationary, (b) location traces for user B where he walked around his office, and (c) walking traces of user A from his house to the bus stop.

Regarding clustering physical activities, manually tracked activities are clustered based on the type of activities similar to food clustering. Automatically tracked activities, tagged with location information, are clustered by places they occur. Clusters are found using unsupervised machine-learning techniques to identify similarity.  As indoor localization is often accurate up to 150 meters, any stationary activities that fall within 150 meters of each other are clustered together.  For example, a user’s stationary activities in the office are typically in close proximity to each other and, as such, MyBehavior clusters these office locations into a single unit that represents the user’s stationary behavior in the office.  Walking and running activities are more difficult to cluster because MyBehavior needs to determine whether two activity trajectories look similar and happen at a similar location.  To group similar walking or running events, MyBehavior uses an algorithm derived from the literature on handwriting recognition [27]. In handwriting recognition, the task is to find a canonical letter that matches the shape or trajectory of a handwritten letter.  The analogous task in MyBehavior is to find whether a new walking trajectory (eg, office to coffee shop) matches previous walking trajectories. Figure 3 shows some clusters generated by this technique.  The image on the left represents a user’s stationary episode in the office and home, whereas the middle and right-hand images show two walking clusters generated by two different users. The middle image represents a user’s walks near the office, while the cluster in the right-hand image represents another user’s daily walks from home to a bus stand.  

#### Suggestion Generation

After clustering user behaviors, MyBehavior uses an *exploit-explore strategy* to automatically generate suggestions based on users’ past physical activities and food intake. This suggestions-generating strategy is grounded in contemporary behavioral science theories:  (1) learning theory [28], (2) social cognitive theory [29], and (3) the Fogg Behavior Model (FBM) [30].  Behavior analysis applies learning theory first to assess whether a person has the skills needed to perform a behavior [28]. If so, the next step is to increase or decrease the target behavior’s frequency by harnessing its antecedents (ie, its setting and cues) and consequences (ie, reinforcement). For example, if a health suggestion asks a user to swim but the user can’t swim (ie, he never acquired the skills), the user will not follow the suggestion. On the other hand, if a person has performed a behavior before, even if rarely, the skills can be assumed present. The Fogg Behavior Model applies theoretical principles to technology design by creating tools to prompt *low-effort* actions that can be triggered even when motivation is low [30].   Thus, MyBehavior suggests (ie, cues or triggers) a frequent behavior (eg, a particular walk) that the person often does in a particular life context. This small, low-effort change simply increases the frequency of a behavior that the person already does. Sometimes instead, MyBehavior suggests an infrequent behavior (eg, bike ride) that would burn more calories and that the person has shown he/she can do, but does only rarely. Social cognitive theory [29], the most widely used behavioral theory, suggests that in order to voluntarily initiate an action, a person needs a sense of self-efficacy or confidence that he/she will be able to perform it.  The more frequently the person can be triggered to ride a bike *repeatedly* in a certain *context* where bikes are accessible, the more self-efficacy increases, the less effortful the behavior becomes, and the more likely that bike riding becomes a habit.

MyBehavior *exploits* the frequency principle by suggesting activities that users perform repeatedly. In addition, the algorithm favors actions that are not only frequent, but also result in higher calorie expenditure. For example, short 1-minute walks inside the office, though very frequent, are likely to be superseded in the suggestion-generation engine by a less frequent, but higher-calorie-burning, gym class. On the other hand, if the person rarely visits the gym but walks 30 minutes to work several times a week, the recommender engine will rank the walk higher than the gym since the aggregate calorie loss—*frequency x calories* burned each instance—is higher.

**Figure 4:**
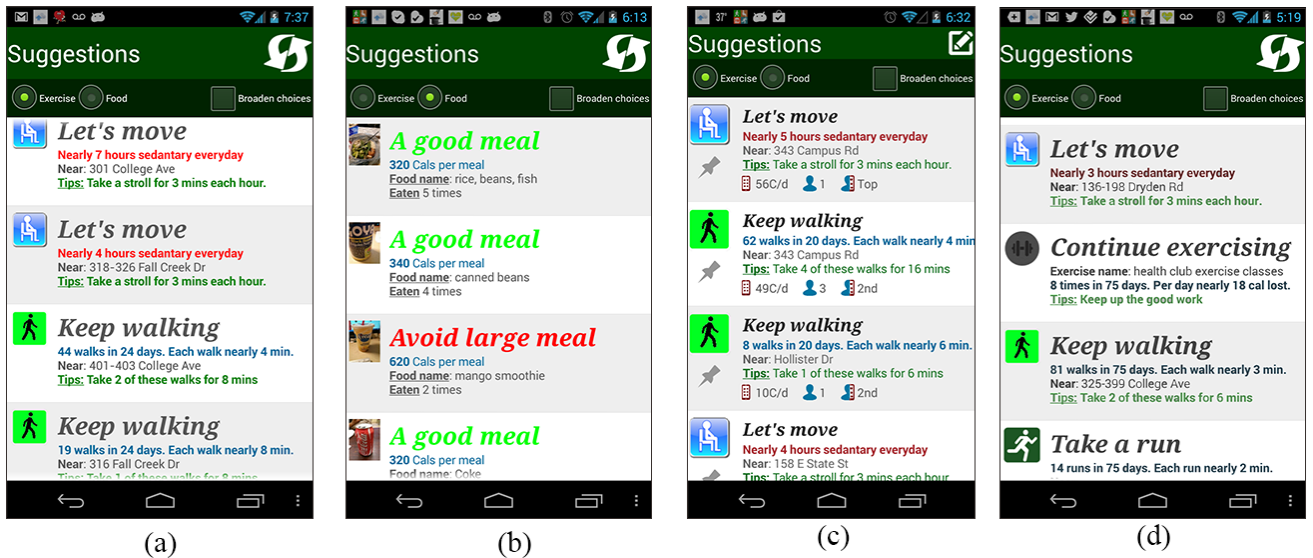
Screenshots showing recommended suggestions for exercise and food: (a) physical activity suggestions made by MyBehavior, (b) food suggestions made by MyBehavior, (c) physical activity suggestions for a different user, and (d) physical activity suggestions for the same user as in (a), but at a different point in time.

For stationary activities, the recommender engine suggests small changes, such as walking 3 minutes for every hour spent stationary. The right-most image in Figure 4 shows a prioritization order of MyBehavior suggestions where simply adding 3-minute walks to the user’s hour-long stationary episodes burns more calories compared to rarely occurring gym visits.

*Exploit* suggestions generated solely from users’ frequent past behavior may not generate sufficient energy expenditure to cause weight loss. Consequently, MyBehavior periodically suggests higher-calorie-burning activities to entice the user to try out and adopt.  *Explore* suggestions target infrequent, high-calorie-burning behaviors that the user can turn into a more regular activity.  Future behavior is only imperfectly predicted by past behavior, and it could be the case that users will increase infrequent activities if suggested. Hence, if a user walks regularly near her office but sometimes goes to the gym or takes a long walk home, MyBehavior exploits this knowledge by suggesting walking near the office most of the time and by sometimes suggesting a gym visit or a long walk. If the new suggestion sticks and the user starts going to the gym regularly as a result, then MyBehavior learns to target the gym as an exploit suggestion rather than an explore suggestion.

When generating food suggestions, a separate set of suggestions is created based on the exploit-explore strategy. First, MyBehavior distinguishes between meals and snacks. Then it takes into account both intake frequency and calories similar to the physical activity suggestions. Thus, a user’s frequent healthy low-calorie meals are exploited and are encouraged to be continued. During exploration, a random selection of infrequent low-calorie meals/snacks from the past is suggested. Here, the expectation is that users will take up some of these infrequent meals and make them frequent in the future.  

At the start of every day, MyBehavior generates 10 food and 10 activity suggestions.  Of these, 90% are from the users’ most frequent activities (ie, exploit) and 10% are from the users’ infrequent behaviors (ie, explore). This split of 90% exploit and 10% explore was heuristically chosen based on previous literature [31]. This kind of exploit-explore strategy, well grounded in artificial intelligence research, falls under a wider decision-making framework called multi-armed bandit [31]. MAB models have been well studied for modeling dynamic systems where situations can change over time. In our case, user behavior is not fixed and can change over time under MyBehavior’s influence (see Figure 4, left-most and right-most images). The exploit-and-explore strategy models this dynamic nature of human behavior effectively.  MyBehavior exploits the most common user behaviors that promote energy balance to produce short-term health gain. To target long-term health, it occasionally explores infrequent higher-energy-expending behaviors to discover actions that the user might repeat in the future, leading to sustained energy balance that could boost weight loss.

Figure 4 shows different generated suggestions that encourage the user to either continue positive activities (ie, low-calorie foods, walking, or exercise), make small changes in some situations (ie, stationary activities) (left-most image), or avoid negative activities (ie, frequent large meals) (second image). The first and third images in Figure 4 show suggestions for two different users and the first and last images show suggestions for the same user that change over time.  A video demonstrating different features of MyBehavior is included as Multimedia Appendix 2.

### Measures

First, we used a suggestion-rating survey to evaluate user *intentions* to follow the suggestions. Participants completed this survey after the 3-week study concluded. Participants rated the suggestions, by indicating on a 1-to-5 scale, whether they would be willing and able to do the recommended action on an average day—5 (Strongly Agrees that he/she can follow the suggestion), 1 (Strongly Disagrees). Each participant rated suggestions that she/he saw during the study in an online form. Experimental group participants rated 15 top-ranked—top 8 physical activity and top 7 food—personalized MyBehavior suggestions of their own. On the other hand, the control group participants rated 10 randomly chosen generic prescriptive suggestions. In addition, we quantitatively measured *behavior change* for all participants using logs of daily physical activity and dietary intakes.

The daily diary and the in-depth, semistructured interviews measured participant feedback regarding the suggestions. For the daily diaries, we queried (1) whether they looked at MyBehavior’s suggestions, and (2) whether they made or wanted to make any changes after seeing the suggestions. The semistructured interviews covered users’ general overall experience with MyBehavior and the quality of the suggestions. Specifically, we inquired about awareness, behavior change, and of any software improvement they would like to see. In addition, in the interview, we asked clarifying questions that explained quantitative results observed from the data.

### Analysis Plan

Regarding the user’s intention of following MyBehavior’s suggestions, we gathered ratings for suggestions on a secure website and analyzed the data using RStudio. Since the ratings were in ordinal scale, we used a nonparametric Mann-Whitney U test [32] for statistical significance and effect size.

We measured behavior changes by analyzing activity and dietary logs for statistical significance using MATLAB (MathWorks, Inc) statistics toolbox and RStudio. For each user, we computed median walking length and calories per food item. We considered medians across entire weeks over other central measures since they are less susceptible to spurious noise or outliers (eg, occasional intake of very-high-calorie food or atypical, unusually lengthy walk). We did not report changes in running and manually logged exercises in the data analysis as they often require higher effort and are tough to change within the 3 weeks of the experiment. In our analysis, we first considered the number of positive changes. A *positive change* is defined as a downward trend in median calories in meals, or an upward trend toward longer-length walks over the first week to the third week. We used the Fisher Exact Test [32] to measure the number of positive changes as an effect of MyBehavior. Because of small sample size, the Fisher Exact Test is used instead of the chi-square test for independence. We used a two-sample independent Student’s *t* test to measure statistical significance for total walk lengths and total food calories consumed per day. We computed differences in walking distances instead of total number of calories burned, since a walk of a fixed distance can result in a different amount of calories burned for different individuals [25]. We calculated the effect size of walking and eating behavior changes with Cohen’s d measure.

Finally, face-to-face, semistructured interviews were audio recorded and transcribed. Interview transcripts and daily diaries were then broken down into themes using thematic analysis [33].

## Results

### Adherence

A total of 17 participants completed the 3-week study, yielding almost 2.1 million recorded physical activity instances, amounting to more than 8000 hours of physical activity. During the same period, participants labeled nearly 850 images of food with annotations.

### User Acceptance of MyBehavior Suggestions

In the suggestion-rating survey, the experimental group (mean 3.4, SD 1.2, q_25_=2.75, q_50_=3, q_75_=4), with MyBehavior suggestions, intended to follow personalized suggestions more than the control group (mean 2.5, SD 1.6, q_25_=1, q_50_=2, q_75_=4) intended to follow the generic suggestions. A nonparametric Mann-Whitney U test [32] found this difference to be statistically significant (*P*<.001, 95% CI 0-1.001, effect size = 0.99).

### Physical Activity

**Figure 5:**
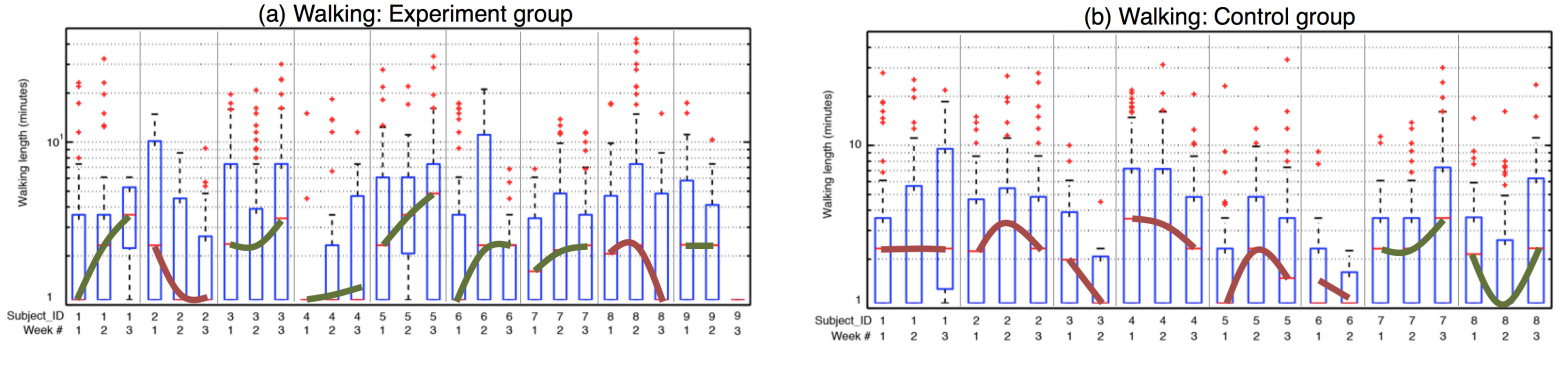
Box plots showing the distribution of walking lengths for the experimental group (a) and for the control group (b) over the 3-week study. We joined the medians of distributions and showed each trend as a thick green line (increasing trend) or red line (decreasing trend) for walking length.

Figure 5 shows the distribution, in the form of box plots, of walking lengths over time for the experimental (left-hand image) and the control (right-hand image) groups. For each week of the study, we computed these distributions for the different users. To ease interpretation, we joined the median per week with thick green or red lines for each user. A green line implies a positive change as discussed in the data analysis section. A red line indicates the reverse negative trend. We used a log scale for walking-length distribution since walking-length distributions have heavy tails [34].

For walking, 78% (7/9) of participants in the experimental group (Figure 5, left-hand image) showed positive trends, whereas 75% (6/8) of participants in the control group (Figure 5, right-hand image) exhibited negative trends.  A Fisher Exact Test found this ratio in the number of positive changes between the experimental and control groups statistically significant (*P*=.05) [35]. In addition, MyBehavior users walked an average of 10 minutes more per day within the experiment phase (ie, from the first to the third week). However, we did not observe any change for the control group. A two-sample *t* test found this difference in change of walking duration to be significant (*t*_15_= 2.1, *P*=.055, 95% CI -0.23 to 19.052, *d*=0.9).

Qualitative data from daily diary and face-to-face interviews largely supported this quantitative result. However, we also observed some important subtleties. First, participants in the experimental group described the activity suggestions to be actionable and relevant to their lives. Control group participants appreciated that the generic suggestions reminded them of good habits. However, they often faced problems incorporating the suggestions into their daily lives. The following quotes were taken from the daily diaries of participants.

Those suggestions are quite good, which reminds me not to sit too long in one place.

Experimental group participant #1

The exercise suggestions made me want to do some more activities and be less stationary. Seeing how long I have been stationary and the low frequency of activity made me want to make a change.

Experimental group participant #5

Try to get up from my desk more often...added “walk" notes to my calendar.

Experimental group participant #2

I did some walking where I normally walk. The app now shows I walked there 26 times. The app makes me feel that I can do it again since I have done the same walk many times.

Experimental group participant #7

The suggestions encourage me to do/plan exercises for the near future...It reminds me that some foods are better than others.

Control group participant #1

They seem like good generic suggestions. The kind you would read...as tips in a health magazine or some such...

Control group participant #4

Some MyBehavior users reported that even the nonfrequent explore suggestions were actionable and expressed interest in acting on them. For instance, experimental group participant #7 said the following in his/her daily diary:

I saw a walk to my nearest bus stand listed. Normally, I drive my car to go to my office. But looking at the extra walking I got while going to the bus stop makes me think about doing it often and making it a habit.

Experimental group participant #7

Results from interviews also revealed that participants at various stages of active lifestyle reacted to suggestions differently [15]. For the experimental group, participants who were considering making changes expressed that they became more self-conscious about their behavior and they were eager to follow the suggested changes (eg, starting to walk more near home, or continuing runs on treadmills). Comparatively, users likely maintaining an active lifestyle expressed that the suggestions reflected their current healthy behavior and considered them as good reinforcements. However, participants in the maintenance phase wanted to change their stationary behavior in the office with occasional small walks. For the control group, users were frustrated because the suggestions were not always feasible and did not blend with their routines and lifestyle. Control group users maintaining an active lifestyle were unaffected by generic suggestions and continued their regular behavior across weeks. For example, control group participants #7 and #8 were maintaining participants and their behavior showed no negative trends in Figure 5 (right-hand image). Control group users who did not already have a maintaining lifestyle gradually became less active or made poorer food choices after the initial phase of the study.

Finally, on a few occasions, MyBehavior suggestions were hard to follow or did not reflect user preferences. For example, one user reported in the interview that he used to play soccer with his friends but his friends recently moved to a new location. He could no longer play soccer, which MyBehavior was suggesting. In addition, often user-preferred activities are not top MyBehavior suggestions. For instance, one user preferred to swim even though she did not do it often. Finally, experimental group participant #8 (subject 8 in Figure 5, left-hand image, with negative trends) reported an inability to follow MyBehavior suggestions because of a looming work deadline during the study.

### Dietary Behavior

**Figure 6:**
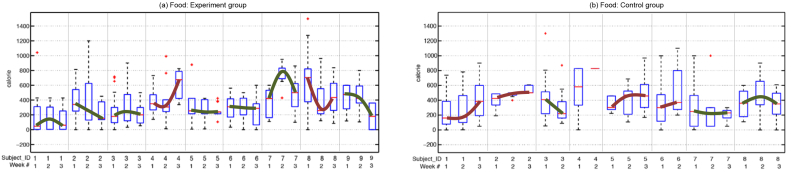
Box plots showing the distribution of food calories for the experimental group (a) and for the control group (b) over the 3-week study. We joined the medians of distributions and showed each trend as a thick green line (increasing trend) or red line (decreasing trend) for median food calorie intake.

Figure 6 shows the distribution, in the form of box plots, of meal calories for the experimental group (left-hand image) and the control group (right-hand image). For each week of the study, we computed these distributions for different users. Similar to walking-behavior graphs, we joined medians across weeks to show positive or negative changes for each user.

For caloric intake, 78% (7/9) of participants in the experimental group showed positive trends (green lines in Figure 6, left-hand image), and 57% (4/7) of participants in the control group showed negative trends (red lines in Figure 6, right-hand image—1 participant had insufficient data). However, a Fisher Exact Test found this to be nonsignificant (*P*=.15). For control group participants, we also found their average median calories per day to increase by 211 calories (mean 211.7, SD 263.07, q_25_=-31.25, q_50_=187.5, q_75_=429.35) from the first week to the third week. Comparatively, the experimental group showed an average calorie per day decrease of nearly 100 calories (mean -99.3, SD 481.27, q_25_=-527.83, q_50_=-37.3, q_75_=87.5) from the first week to the third week. This change was not significant in a two-sample *t* test (*t*_12_=1.3234, *P*=.21, 95% CI -201 to 822.96, *d*=0.72).

In qualitative feedback, similar to physical activity suggestions, experimental group users found the suggestions to be more actionable and reported to make more changes compared to control group users who found the suggestions to be hard to work on. This feedback is illustrated in the following quotes from participants’ daily diaries.

The pictures of my meals are very useful to keep track of what I've been eating in the past. People tend to forget about their habits, but pictures in this case are a nice way to bring your food history in front of your eyes.

Experimental group participant #9

The suggestions remind me that some foods are better than others.

Control group participant #1

It recommends me to eat stuff that I don't have at home.

Control group participant #4

These suggestions don't take into account my dietary restrictions.

Control group participant #5

Similar to activity explore suggestions, MyBehavior users often found the explore suggestions to be actionable.

I just wanted to see what it was...These ones [explore suggestions] seemed to pick up some "good" food habits.

Experimental group participant #4

Finally, users reported manual food logging to be time consuming in the interview. However, they also reported that this manual process made them more aware of their foods. Consequently, control group participants reported making dietary changes without personalized suggestions.

## Discussion

### Principal Findings

To our knowledge, MyBehavior is the first system to automatically provide personalized suggestions that relate to users’ lifestyles. In the quantitative results, MyBehavior users demonstrated superior behavior changes compared to the control group. Qualitative measures from the face-to-face interviews and the daily diaries confirmed that the suggestions indeed were perceived to be personalized to their lives. This concordance of superiority in both quantitative behavior change and qualitative user perception makes MyBehavior’s automated health feedback approach very promising and provides support for longitudinal studies and future investigations into automated personalization approaches.

Specifically, in our evaluation, users rated that they could follow MyBehavior personalized suggestions more than the control condition suggestions. Results also revealed a significant change in walking behaviors for MyBehavior users. In qualitative measures, users reported MyBehavior activity suggestions to be more actionable. Interestingly, although users qualitatively reported the dietary suggestions to be more actionable, dietary behavior changes were not found to be different between the groups. This finding could be due to the manual-logging nature of food intake being sufficient for behavior change alone. The manual process of food logging might produce self-awareness and reflection. Indeed, past research demonstrates that simple logging can improve one’s food consumption behavior [16]. However, food logging is an arduous process and it is often hard to continue for an extended period. Thus, we need longer studies to determine if food logging along with suggestions could aid in sustained behavior change. Furthermore, we had a small sample in the study with inadequate statistical power. Thus, larger trials are necessary to further elucidate the effects of food logging and these types of suggestions on eating behavior.

Nonetheless, MyBehavior explores a unique space for health feedback. Earlier studies in this domain predominantly focused on overall behavior [7,14], tailoring [36], or self-tracking [33] without deeper data analysis and personalization. MyBehavior takes a data mining approach to automatically find contextualized suggestions from logged data. This automated approach also relieves users from the burden of self-analyzing their data. Thus, MyBehavior is a marked departure from previous self-monitoring programs found in the literature, where users themselves decide on how to make changes on their own [33]. MyBehavior suggestions relate to a user’s existing behaviors, making them actionable as the user is told *where* and *when* to act on them. Furthermore, unique sets of suggestions are generated for each user based on their routine and lifestyle. The literature on N-of-1 approaches [37,38,39] argue that such personalization should yield better efficacy than one-size-fits-all or tailored-suggestion approaches [15], where similar suggestions are provided to users with similar characteristics (eg, age, gender, daily calorie intake, and loss).

Despite this promising direction, the automated data-driven personalization approach of MyBehavior brings its own challenges. Manual logging of food and exercise, in addition to automated logging, are necessary for proper functioning of MyBehavior. Qualitative interviews revealed that manual food and exercise logging were often burdensome. Future iterations could use crowdsourcing-based semiautomated approaches to decrease the burden of manual food journaling [12]. Finally, interviews also highlighted the importance of considering contextual changes in users’ lives and preferences. Thus, giving users control in deciding which suggestion they want to follow is required for well-accepted personalization [7].

### Limitations

An important limitation is the short-term and small-scale nature of the study, which makes it difficult to make definitive conclusions. However, the study helped us to identify the potential efficacy of MyBehavior and pinpoint design improvements for future deployments. Indeed, Klasanja et al [17] argued that such short-term studies with similar evaluation goals as in our study are often more suitable for new and untested behavior change technologies like MyBehavior. Another limitation was that the nonpersonalized suggestions were sometimes too specific, for example, “walking with a dog.” In the daily diaries, some users reported that they could not follow this suggestion since they did not own a dog. While designing generic suggestions, we tried to find suggestions that most users could follow, without being overly generic. However, there will always be exceptions where a suggestion does not fit one’s lifestyle.

Despite these limitations, this pilot study demonstrates the potential of using automated personalization for actionable health feedback. As we move into an age where increasingly more people are tracking their health with mobile and other technologies, we believe MyBehavior’s automated technique holds great potential to provide feedback that can be used to improve health outcomes at scale.

### Conclusions

MyBehavior is the first mobile health app that can encourage healthy behavior change by automatically providing low-effort suggestions based on the context and personal information of users.  The pilot user study demonstrated the feasibility and acceptability of MyBehavior. Users considered MyBehavior’s personalized, contextualized suggestions to be more actionable and to require less effort to implement than generic prescriptive suggestions. Preliminary evidence of behavior change shows that a high percentage of MyBehavior users did more physical exercise, yet the potential impact on eating behaviors remains unclear. The addition of more human control over the suggestions and providing easier logging mechanisms for food and exercise were identified as key areas of improvement. Future directions should include addressing the identified shortcomings of the system and testing its effectiveness in fostering health behavior change in a larger longitudinal trial.

## Appendix

Multimedia Appendix 1 Generic suggestions used in the control group.

Multimedia Appendix 2 A video showing how MyBehavior works.

Multimedia Appendix 3 CONSORT-EHEALTH checklist V1.6.1 [40].

## Abbreviations

CDC: Centers for Disease Control and Prevention
FBM: Fogg Behavior Model
GMM: Gaussian Mixture Model
GPS: Global Positioning System
MAB: multi-armed bandit
METS: Metabolic Equivalents of Task
q25: lower quartile
q50: median
q75: upper quartile
RCT: randomized control trial
USDA: United States Department of Agriculture
WHO: World Health Organization
