# Supplementary material for: Automated Personalized Feedback for Physical Activity and Dietary Behavior Change With Mobile Phones: A Randomized Controlled Trial on Adults
Source: JMIR Mhealth Uhealth. 2015 May 14;3(2):e42. doi: 10.2196/mhealth.4160 (PMC4812832; doi:10.2196/mhealth.4160)
Supplement: Multimedia Appendix 1 [file mhealth_v3i2e42_app1.pdf]

Summary of all the generic suggestions:

Food suggestions:

1. Snack on a handful of nuts, e.g. almonds (20ish)
2. eat eggs (hardboiled, poached, scrambled) for any meal or snack
3. oatmeal for breakfast
4. put mustard instead of mayonnaise on sandwich
5. use a whole wheat wrap instead of bread for a sandwich
6. snack on baby carrots or celery sticks with hummus
7. apple with nut butter for a snack
8. eat soup (clear broth not creamy)
9. small piece of lowfat cheese or a string cheese for a snack
10. try sweet potatoes instead of white potatoes
11. snack on salsa and baked tortilla chips
12. put lowfat turkey and lots of veggies on a sandwich
13. wholegrain pretzels for a snack
14. snack on a rice cake with hummus or nut butter
15. whole wheat english muffin with nut butter for breakfast
16. eat fresh fruit (apple, banana, orange, pear, plum, red grapes, kiwi, berries)
17. eat whole fruit instead of drinking fruit juice
18. use skim milk instead of halfandhalf or whole milk in coffee
19. low fat greek yogurt for breakfast, lunch or a snack
20. cottage cheese for a snack or lunch
21. snack on airpopped or light microwave popcorn
22. drink green tea or water instead of soda
23. salad with light dressing for lunch
24. steam or roast your vegetables (broccoli, squash, asparagus, etc)
25. shrimp or other shellfish for dinner
26. skinless chicken breast for dinner
27. fish for dinner
28. lean meat (pork tenderloin, lean beef, etc) for dinner
29. replace meat with tofu, tempeh or seitan
30. eat beans, lentils, or chickpeas instead of meat
31. wholewheat pasta with red sauce and veggies for dinner
32. eat brown rice instead of white

Exercise suggestions:

1. Walk 30 minutes
2. Add intervals: walk 5 minutes, run 5 minutes, repeat 3 times
3. Take the stairs instead of the elevator whenever possible
4. Take a dog for a walk
5. Swim a lap, rest for 1 minute, repeat 510 times
6. Try a fitness class at the gym

7. Strength training bodyweight exercises like pushups, tricep dips, squats, lunges, planks
8. Yoga
9. Park at the far end of the parking lot to walk further
10. Yardwork
