# Supplementary material for: Automated Personalized Feedback for Physical Activity and Dietary Behavior Change With Mobile Phones: A Randomized Controlled Trial on Adults
Source: JMIR Mhealth Uhealth. 2015 May 14;3(2):e42. doi: 10.2196/mhealth.4160 (PMC4812832; doi:10.2196/mhealth.4160)
Supplement: Multimedia Appendix 3 [file mhealth_v3i2e42_app3.pdf]

CONSORT-EHEALTH (V 1.6.1) - Submission/Publication Form

The CONSORT-EHEALTH checklist is intended for authors of randomized trials evaluating web-based and Internet-based applications/interventions, including mobile interventions, electronic games (incl multiplayer games), social media, certain telehealth applications, and other interactive and/or networked electronic applications. Some of the items (e.g. all subitems under item 5 - description of the intervention) may also be applicable for other study designs.

The goal of the CONSORT EHEALTH checklist and guideline is to be
a) a guide for reporting for authors of RCTs,
b) to form a basis for appraisal of an ehealth trial (in terms of validity)

CONSORT-EHEALTH items/subitems are MANDATORY reporting items for studies published in the Journal of Medical Internet Research and other journals / scientific societies endorsing the checklist.

Items numbered 1., 2., 3., 4a., 4b etc are original CONSORT or CONSORT-NPT (non-pharmacologic treatment) items.
Items with Roman numerals (i., ii, iii, iv etc.) are CONSORT-EHEALTH extensions/clarifications.

As the CONSORT-EHEALTH checklist is still considered in a formative stage, we would ask that you also RATE ON A SCALE OF 1-5 how important/useful you feel each item is FOR THE PURPOSE OF THE CHECKLIST and reporting guideline (optional).

Mandatory reporting items are marked with a red *.
In the textboxes, either copy & paste the relevant sections from your manuscript into this form - please include any quotes from your manuscript in QUOTATION MARKS,
or answer directly by providing additional information not in the manuscript, or elaborating on why the item was not relevant for this study.

YOUR ANSWERS WILL BE PUBLISHED AS A SUPPLEMENTARY FILE TO YOUR PUBLICATION IN JMIR AND ARE CONSIDERED PART OF YOUR PUBLICATION (IF ACCEPTED).
Please fill in these questions diligently. Information will not be copyedited, so please use proper spelling and grammar, use correct capitalization, and avoid abbreviations.

DO NOT FORGET TO SAVE AS PDF _AND_ CLICK THE SUBMIT BUTTON SO YOUR ANSWERS ARE IN OUR DATABASE !!!

Citation Suggestion (if you append the pdf as Appendix we suggest to cite this paper in the caption):
Eysenbach G, CONSORT-EHEALTH Group
CONSORT-EHEALTH: Improving and Standardizing Evaluation Reports of Web-based and Mobile Health Interventions
J Med Internet Res 2011;13(4):e126
URL: http://www.jmir.org/2011/4/e126/
doi: 10.2196/jmir.1923
PMID: 22209829

* Required

Your name *

First Last

Mashfiqui Rabbi

Primary Affiliation (short), City, Country *

University of Toronto, Toronto, Canada

Cornell University

Your e-mail address *

abc@gmail.com

ms2749@cornell.edu

Title of your manuscript *

Provide the (draft) title of your manuscript.

Automated Personalized Feedback for Physical Activity and Dietary Behavior Change with Smartphones: Development and a Mixed-Method Feasibility Study on Adults

Article Preparation Status/Stage *

At which stage in your article preparation are you currently (at the time you fill in this form)

- ○ not submitted yet - in early draft status
- ○ not submitted yet - in late draft status, just before submission
- ○ submitted to a journal but not reviewed yet
- ● submitted to a journal and after receiving initial reviewer comments
- ○ submitted to a journal and accepted, but not published yet
- ○ published
- ○ Other:

Journal *

If you already know where you will submit this paper (or if it is already submitted), please provide the journal name (if it is not JMIR, provide the journal name under "other")

- ○ not submitted yet / unclear where I will submit this
- ● Journal of Medical Internet Research (JMIR)
- ○ Other:

Manuscript tracking number *

If this is a JMIR submission, please provide the manuscript tracking number under "other" (The ms tracking number can be found in the submission acknowledgement email, or when you login as author in JMIR. If the paper is already published in JMIR, then the ms tracking number is the four-digit number at the end of the DOI, to be found at the bottom of each published article in JMIR)

○ no ms number (yet) / not (yet) submitted to / published in JMIR

◉ Other: 4160

TITLE AND ABSTRACT

1a) TITLE: Identification as a randomized trial in the title

1a) Does your paper address CONSORT item 1a? *

I.e does the title contain the phrase "Randomized Controlled Trial"? (if not, explain the reason under "other")

○ yes

◉ Other:

1a-i) Identify the mode of delivery in the title

Identify the mode of delivery. Preferably use “web-based” and/or “mobile” and/or “electronic game” in the title. Avoid ambiguous terms like “online”, “virtual”, “interactive”. Use “Internet-based” only if Intervention includes non-web-based Internet components (e.g. email), use “computer-based” or “electronic” only if offline products are used. Use “virtual” only in the context of “virtual reality” (3-D worlds). Use “online” only in the context of “online support groups”. Complement or substitute product names with broader terms for the class of products (such as “mobile” or “smart phone” instead of “iphone”), especially if the application runs on different platforms.

	1	2	3	4	5	
subitem not at all important	○	○	○	○	◉	essential

Does your paper address subitem 1a-i? *

Copy and paste relevant sections from manuscript title (include quotes in quotation marks "like this" to indicate direct quotes from your manuscript), or elaborate on this item by providing additional information not in the ms, or briefly explain why the item is not applicable/relevant for your study

Automated Personalized Feedback for Physical Activity and Dietary Behavior Change with Smartphones: Development and a Mixed-Method Feasibility Study on Adults

1a-ii) Non-web-based components or important co-interventions in title

Mention non-web-based components or important co-interventions in title, if any (e.g., “with telephone support”).

	1	2	3	4	5	
subitem not at all important	○	○	○	○	◉	essential

Does your paper address subitem 1a-ii?

Copy and paste relevant sections from manuscript title (include quotes in quotation marks "like this" to indicate direct quotes from your manuscript), or elaborate on this item by providing additional information not in the ms, or briefly explain why the item is not applicable/relevant for your study

“This item is not applicable as all intervention components were delivered via the smartphone application.”

1a-iii) Primary condition or target group in the title

Mention primary condition or target group in the title, if any (e.g., “for children with Type I Diabetes”) Example: A Web-based and Mobile Intervention with Telephone Support for Children with Type I Diabetes: Randomized Controlled Trial

	1	2	3	4	5	
subitem not at all important	○	○	○	○	◉	essential

Does your paper address subitem 1a-iii? *

Copy and paste relevant sections from manuscript title (include quotes in quotation marks "like this" to indicate direct quotes from your manuscript), or elaborate on this item by providing additional information not in the ms, or briefly explain why the item is not applicable/relevant for your study

"Automated Personalized Feedback for Physical Activity and Dietary Behavior Change with Smartphones: Development and a Mixed-Method Feasibility Study on Adults"

1b) ABSTRACT: Structured summary of trial design, methods, results, and conclusions

NPT extension: Description of experimental treatment, comparator, care providers, centers, and blinding status.

1b-i) Key features/functionalities/components of the intervention and comparator in the METHODS section of the ABSTRACT

Mention key features/functionalities/components of the intervention and comparator in the abstract. If possible, also mention theories and principles used for designing the site. Keep in mind the needs of systematic reviewers and indexers by including important synonyms. (Note: Only report in the abstract what the main paper is reporting. If this information is missing from the main body of text, consider adding it)

	1	2	3	4	5	
subitem not at all important	○	○	○	○	◉	essential

Does your paper address subitem 1b-i? *

Copy and paste relevant sections from the manuscript abstract (include quotes in quotation marks "like this" to indicate direct quotes from your manuscript), or elaborate on this item by providing additional information not in the ms, or briefly explain why the item is not applicable/relevant for your study

"We have built a smartphone application, MyBehavior, that: (1) uses a combination of automatic and manual logging of user physical activities (e.g. walking, running, gym), user location and food; (2) automatically analyzes activity and food logs to identify frequent and non-frequent behaviors; and (3) uses a standard machine learning decision-making algorithm, called multi-armed bandit (MAB), to generate personalized suggestions that ask users to either continue, avoid or make small changes to existing behaviors in order to help users reach their calorie goals.

We enrolled 17 participants, all motivated to self-monitor and improve their fitness, in a pilot study of MyBehavior. In a two-arm study design, participants were randomly assigned to receive either MyBehavior's personalized suggestions (n=9) or non-personalized suggestions created by professionals (n=8) over 3 weeks. Participants maintained daily diaries and were interviewed regarding their experiences with MyBehavior. Daily activity level and dietary intakes were monitored from tracked data. At the end of study, a survey was conducted that asked users to subjectively rate their intention to follow MyBehavior suggestions. Participants were financially incentivized (80 dollars for three weeks) to promote adherence during the study."

1b-ii) Level of human involvement in the METHODS section of the ABSTRACT

Clarify the level of human involvement in the abstract, e.g., use phrases like "fully automated" vs. "therapist/nurse/care provider/physician-assisted" (mention number and expertise of providers involved, if any). (Note: Only report in the abstract what the main paper is reporting. If this information is missing from the main body of text, consider adding it)

	1	2	3	4	5	
subitem not at all important	○	○	○	○	◉	essential

Does your paper address subitem 1b-ii?

Copy and paste relevant sections from the manuscript abstract (include quotes in quotation marks "like this" to indicate direct quotes from your manuscript), or elaborate on this item by providing additional information not in the ms, or briefly explain why the item is not applicable/relevant for your study

"We have built a smartphone application, MyBehavior, that: (1) uses a combination of "automatic" and "manual logging" of user physical activities (e.g. walking, running, gym), user location and food; (2) "automatically" analyzes activity and food logs to identify frequent and non-frequent behaviors; and (3) uses a standard machine learning decision-making algorithm, called multi-armed bandit (MAB), to generate personalized suggestions that ask users to either continue, avoid or make small changes to existing behaviors in order to help users reach their calorie goals."

1b-iii) Open vs. closed, web-based (self-assessment) vs. face-to-face assessments in the METHODS section of the ABSTRACT

Mention how participants were recruited (online vs. offline), e.g., from an open access website or from a clinic or a closed online user group (closed usergroup trial), and clarify if this was a purely web-based trial, or there were face-to-face components (as part of the intervention or for assessment). Clearly say if outcomes were self-assessed through questionnaires (as common in web-based trials). Note: In traditional offline trials, an open trial (open-label trial) is a type of clinical trial in which both the researchers and participants know which treatment is being administered. To avoid confusion, use “blinded” or “unblinded” to indicated the level of blinding instead of “open”, as “open” in web-based trials usually refers to “open access” (i.e. participants can self-enrol). (Note: Only report in the abstract what the main paper is reporting. If this information is missing from the main body of text, consider adding it)

	1	2	3	4	5	
subitem not at all important	○	○	○	○	●	essential

Does your paper address subitem 1b-iii?

Copy and paste relevant sections from the manuscript abstract (include quotes in quotation marks "like this" to indicate direct quotes from your manuscript), or elaborate on this item by providing additional information not in the ms, or briefly explain why the item is not applicable/relevant for your study

"We enrolled 17 participants, all motivated to self-monitor and improve their fitness, in a pilot study of MyBehavior. In a randomized two-group trial, participants were randomly assigned by investigators to receive either MyBehavior’s personalized suggestions (n=9) or non-personalized suggestions created by professionals (n=8) over 3 weeks. The suggestions were delivered through the smartphone. Daily activity level and dietary intake was monitored from logged data. At the end of the study, an in-person survey was conducted that asked users to subjectively rate their intention to follow MyBehavior suggestions."

1b-iv) RESULTS section in abstract must contain use data

Report number of participants enrolled/assessed in each group, the use/uptake of the intervention (e.g., attrition/adherence metrics, use over time, number of logins etc.), in addition to primary/secondary outcomes. (Note: Only report in the abstract what the main paper is reporting. If this information is missing from the main body of text, consider adding it)

	1	2	3	4	5	
subitem not at all important	○	○	○	○	●	essential

Does your paper address subitem 1b-iv?

Copy and paste relevant sections from the manuscript abstract (include quotes in quotation marks "like this" to indicate direct quotes from your manuscript), or elaborate on this item by providing additional information not in the ms, or briefly explain why the item is not applicable/relevant for your study

In qualitative daily diary, interview, and survey data, users reported MyBehavior suggestions to be highly actionable and stated that they intended to follow the suggestions. MyBehavior users (n=9) walked significantly more than the control group (n=8) over the three weeks (p=0.05). Although some MyBehavior users chose lower calorie foods the between groups difference was not significant (p=0.15). In post-study survey, users rated MyBehavior's personalized suggestions more positively than the non-personalized generic suggestions created by professionals (p<0.001).

1b-v) CONCLUSIONS/DISCUSSION in abstract for negative trials

Conclusions/Discussions in abstract for negative trials: Discuss the primary outcome - if the trial is negative (primary outcome not changed), and the intervention was not used, discuss whether negative results are attributable to lack of uptake and discuss reasons. (Note: Only report in the abstract what the main paper is reporting. If this information is missing from the main body of text, consider adding it)

	1	2	3	4	5	
subitem not at all important	○	○	○	○	●	essential

Does your paper address subitem 1b-v?

Copy and paste relevant sections from the manuscript abstract (include quotes in quotation marks "like this" to indicate direct quotes from your manuscript), or elaborate on this item by providing additional information not in the ms, or briefly explain why the item is not applicable/relevant for your study

Our primary outcome did change as expected, thus this section is not applicable.

INTRODUCTION

2a) In INTRODUCTION: Scientific background and explanation of rationale

2a-i) Problem and the type of system/solution

Describe the problem and the type of system/solution that is object of the study: intended as stand-alone intervention vs. incorporated in broader health care program? Intended for a particular patient population? Goals of the intervention, e.g., being more cost-effective to other interventions, replace or complement other solutions? (Note: Details about the intervention are provided in “Methods” under 5)

	1	2	3	4	5	
subitem not at all important	○	○	○	○	◉	essential

Does your paper address subitem 2a-i? *

Copy and paste relevant sections from the manuscript (include quotes in quotation marks "like this" to indicate direct quotes from your manuscript), or elaborate on this item by providing additional information not in the ms, or briefly explain why the item is not applicable/relevant for your study

"In 2010, the World Health Organization (WHO) attributed 63% of deaths to non-communicable diseases that are largely preventable [60]. The Centers for Disease Control and Prevention (CDC) estimates that in the U.S. nearly 200,000 deaths annually could be prevented based upon modifications in diet, exercise, and obesity [61]. Obesity alone affects more than one third of the adult population [11] and burdens the U.S. with an estimated $190 billion annually in health care costs [57].

A rapid rise has occurred in the development of smartphone applications and wearable devices to address diet and physical activity. While empirical data is lacking for some commercial applications and sensor-based technologies [15,39], a number of scientific studies have explored the impact of novel technology supported behavior change strategies on physical activity. For example, Weegen et al. [4] applied behavior change theories to design a mobile application that visualized a summary of physical activity logs and gave clinicians feedback to support their promotion of physical activity [18, 50, 51]. Food logging has proved to be more difficult, burdensome, and time-consuming than tracking physical activity. Recent work, however, has attempted to use image-based systems to decrease burden and enhance accuracy in food tracking with some success. [2, 16, 3]. The ubiquity and ever-presence of these devices gives them the potential to perform assessment and intervention in the right place at the right time.

Although these methods show promise, they continue to fall short by not providing context specific, relevant, personalized help at the moment when the individual needs it to make healthier choices. The science of how to present daily physical activity and dietary intake data back to users also has been at a suboptimal state. To date, feedback has been limited to one of three categories (1) overall numeric summaries [18,19,50] (e.g., step counts), (2) tailored suggestions that only adapt to personal characteristics (e.g., age, gender) and overall behavior (e.g., daily calories consumed and burnt) [54] (3) visualizations that incorporate little processing [66]. Simple goals are offered, but without actionable insights on when, where, and how to achieve them. Visualization of large amounts of minimally processed data produces a

[illegible]
related problem: information overload without clear steps to behavior change. Providing personalized, in the moment, actionable guidance that prompts smaller, but more frequent, changes in existing behavior has potential for greater impact. A deeper look into physical activity and dietary intake data can reveal patterns of both healthy and unhealthy behavior that could be leveraged for personalized feedback. With current technologies, this can be achieved automatically, without human interpretation.

Given these observations, MyBehavior was created to address some shortcomings of current mHealth interventions. MyBehavior uses a machine-learning model, multi-armed bandit (MAB), to automatically create contextualized and personalized suggestions based on the individual's physical activity and dietary intake data collected solely from a mobile phone. Moreover, MyBehavior is one of very few mHealth applications designed on the basis of established behavioral theory. As such, the system reflects and incorporates the contemporary state of the behavioral science knowledge about how to foster healthful change. Based on effective behavior change principles, MyBehavior provides low effort suggestions that request small changes to users' existing repeated behaviors. To the best of our knowledge, MyBehavior is the first mHealth application that encourages healthy behavior change by automatically providing low effort suggestions based on the user's context and personal information."

2a-ii) Scientific background, rationale: What is known about the (type of) system

Scientific background, rationale: What is known about the (type of) system that is the object of the study (be sure to discuss the use of similar systems for other conditions/diagnoses, if appropiate), motivation for the study, i.e. what are the reasons for and what is the context for this specific study, from which stakeholder viewpoint is the study performed, potential impact of findings [2]. Briefly justify the choice of the comparator.

	1	2	3	4	5	
subitem not at all important	○	○	○	○	◉	essential

Does your paper address subitem 2a-ii? *

Copy and paste relevant sections from the manuscript (include quotes in quotation marks "like this" to indicate direct quotes from your manuscript), or elaborate on this item by providing additional information not in the ms, or briefly explain why the item is not applicable/relevant for your study

"Contemporary behavioral science theory reflects evidence-based principles derived from several decades of research on behavior change interventions. MyBehavior's design is based on the following theories: (1) learning theory [19], (2) social cognitive theory [23], and (3) the Fogg behavior model (FBM) [26]. Behavior analysis applies learning theory first to assess whether a person has the skills needed to perform a behavior [19]. If so, the next step is to increase or decrease the target behavior's frequency by harnessing its antecedents (its setting and cues) and consequences (reinforcement). For example, if a health suggestion asks a user to swim but the user can't swim (i.e., he never acquired the skills), the user will not follow the suggestion. On the other hand, if a person has performed a behavior before, even if rarely, the skills can be assumed present. Then it may be possible to increase or decrease the behavior's frequency by managing its antecedents and consequences. This is what MyBehavior does. Usually it suggests (cues or triggers) a frequent behavior (e.g., a particular walk) that the person often does in a particular life context. This small, low effort change simply increases the frequency of a behavior that the person already does. Sometimes, instead, MyBehavior suggests an infrequent behavior (e.g., bike ride) that would burn more calories and that the person has shown he/she can do, but does only rarely. Social cognitive theory [23], the most widely used behavioral theory, suggests that in order to voluntarily initiate an action, a person needs a sense of self-efficacy or confidence that he/she will be able to perform it. The more frequently the person can be triggered to ride a bike repeatedly in a certain context where bikes are accessible, the more self-efficacy increases, the less effortful the behavior becomes, and the more likely that bike riding becomes a habit. The Fogg behavior model applies these theoretical principles to technology design by creating tools to prompt low effort actions that can be triggered even when motivation is low [26]."

2b) In INTRODUCTION: Specific objectives or hypotheses

Does your paper address CONSORT subitem 2b? *

Copy and paste relevant sections from the manuscript (include quotes in quotation marks "like this" to indicate direct quotes from your manuscript), or elaborate on this item by providing additional information not in the ms, or briefly explain why the item is not applicable/relevant for your study

"The objective of this study was to evaluate a new behavior change technology, MyBehavior, using a mixed method approach as suggested by others [64]. We focused on (1) whether the users intended to follow the automated MyBehavior suggestions (2) early indications of behavior change empowered by automated suggestions, and (3) participant feedback that could inform user experience and guide future design of automated health feedback systems."

METHODS

3a) Description of trial design (such as parallel, factorial) including allocation ratio

Does your paper address CONSORT subitem 3a? *

Copy and paste relevant sections from the manuscript (include quotes in quotation marks "like this" to indicate direct quotes from your manuscript), or elaborate on this item by providing additional information not in the ms, or briefly explain why the item is not applicable/relevant for your study

"To evaluate the feasibility of MyBehavior, we conducted a small 3-week two-group randomized control trial. We recruited participants through advertisements placed around the Cornell University campus. Recruitment was restricted to participants who owned an Android smartphone and had interest in fitness. Prior to the study, the investigators arranged face-to-face meetings with the participants and acquired their informed consent. Participants also completed a brief survey to provide demographic data and information about their prior experience with mobile technologies and weight loss/fitness applications. All participants attended a training session, where they installed MyBehavior on their primary smartphone and received basic instructions, including how to enter their gender, height, and weight and set up a weekly weight goal (i.e., lose weight, maintain weight, or gain weight). During the first week, users received a daily summary of their activities and food intake. This baseline week was intended to resemble many modern mobile health apps [15, 39] without suggestions on what behaviors to change.

After the first week, the experimenters conducted an in-depth semi-structured interview about their experience to date and then randomized participants into control and experiment groups. Assignment was single blind, as the study participants did not know their condition, while experimenters had full knowledge about the random assignment.

We provided MyBehavior's personalized context-sensitive suggestions to the experimental group while the control group received generic prescriptive recommendations generated from a pool of 42 suggestions for healthy living, such as "walk for 30 minutes" and "eat fish for dinner." A certified fitness professional created these generic suggestions after following National Institute of Health resources [30,31]. An external nutrition counselor also reviewed the suggestions to ensure that they were both healthy and achievable. For the following two weeks, participants continued to log behaviors, received their respective suggestions. During the entire study period, we asked participants to complete web-based daily diaries to better understand their experience in following the suggestions provided. At the conclusion of the three week period, all participants were asked to complete a brief survey about the suggestions provided and were interviewed again about their experience with the application.

This study was approved by Cornell University Institutional Review Board (Protocol #: 1302003617) and a protocol was registered at clinicaltrails.gov (Protocol ID# NCT02359981).

We recruited 18 participants, 17 of which completed the study: 13 students and 4 professionals, 8 females and 9 males, all between the

ages of 18 and 49 (μ=28.3; σ = 6.96; q25 = 22; q50 = 26.3; q75 = 36). All participants reported low-to-moderate levels of physical activity. The majority of participants were experienced smartphone users; nine participants had previous experience using a food diary, and 6 participants had previously kept an exercise log. After the randomization, participants in the groups were similar in terms of level of active lifestyle and experience with using mobile based self-management tools.

Our sample size was determined based on earlier literature [63, 64, 67] suggesting that small studies (n ≥ 4) are more suitable to test early feasibility of novel behavior change technologies like MyBehavior."

3b) Important changes to methods after trial commencement (such as eligibility criteria), with reasons

Does your paper address CONSORT subitem 3b? *

Copy and paste relevant sections from the manuscript (include quotes in quotation marks "like this" to indicate direct quotes from your manuscript), or elaborate on this item by providing additional information not in the ms, or briefly explain why the item is not applicable/relevant for your study

Not applicable

3b-i) Bug fixes, Downtimes, Content Changes

Bug fixes, Downtimes, Content Changes: ehealth systems are often dynamic systems. A description of changes to methods therefore also includes important changes made on the intervention or comparator during the trial (e.g., major bug fixes or changes in the functionality or content) (5-iii) and other "unexpected events" that may have influenced study design such as staff changes, system failures/downtimes, etc. [2].

	1	2	3	4	5	
subitem not at all important	○	○	○	○	●	essential

Does your paper address subitem 3b-i?

Copy and paste relevant sections from the manuscript (include quotes in quotation marks "like this" to indicate direct quotes from your manuscript), or elaborate on this item by providing additional information not in the ms, or briefly explain why the item is not applicable/relevant for your study

Not applicable.

4a) Eligibility criteria for participants

Does your paper address CONSORT subitem 4a? *

Copy and paste relevant sections from the manuscript (include quotes in quotation marks "like this" to indicate direct quotes from your manuscript), or elaborate on this item by providing additional information not in the ms, or briefly explain why the item is not applicable/relevant for your study

"We recruited seventeen participants through advertisements placed around the Cornell University campus. Recruitment was restricted to participants who owned a smartphone and had interest in fitness."

4a-i) Computer / Internet literacy

Computer / Internet literacy is often an implicit “de facto” eligibility criterion - this should be explicitly clarified.

	1	2	3	4	5	
subitem not at all important	○	○	○	○	●	essential

Does your paper address subitem 4a-i?

Copy and paste relevant sections from the manuscript (include quotes in quotation marks "like this" to indicate direct quotes from your manuscript), or elaborate on this item by providing additional information not in the ms, or briefly explain why the item is not applicable/relevant for your study

"The participants owned smartphones and were well-accustomed using smartphones."

4a-ii) Open vs. closed, web-based vs. face-to-face assessments:

Open vs. closed, web-based vs. face-to-face assessments: Mention how participants were recruited (online vs. offline), e.g., from an open access website or from a clinic, and clarify if this was a purely web-based trial, or there were face-to-face components (as part of the intervention or for assessment), i.e., to what degree got the study team to know the participant. In online-only trials, clarify if participants were quasi-anonymous and whether having multiple identities was possible or whether technical or logistical measures (e.g., cookies, email confirmation, phone calls) were used to detect/prevent these.

	1	2	3	4	5	
subitem not at all important	○	○	○	○	◉	essential

Does your paper address subitem 4a-ii? *

Copy and paste relevant sections from the manuscript (include quotes in quotation marks "like this" to indicate direct quotes from your manuscript), or elaborate on this item by providing additional information not in the ms, or briefly explain why the item is not applicable/relevant for your study

"This study was "single blind". i.e., the study subjects didn't know their experiment conditions, but the experimenters had full knowledge about subjects' experiment conditions."

"We provided MyBehavior's personalized context-sensitive suggestions to the experimental group while the control group received generic prescriptive recommendations generated from a pool of 42 suggestions for healthy living, such as "walk for 30 minutes" and "eat fish for dinner." For the following two weeks, participants continued to log behaviors, received their respective suggestions in their smartphones. During the entire study period, we asked participants to complete web-based daily diaries to better understand their experience in following the suggestions provided. At the conclusion of the three week period, all participants were asked to complete a brief survey about the suggestions provided and were interviewed again face-to-face about their experience with the application."

4a-iii) Information giving during recruitment

Information given during recruitment. Specify how participants were briefed for recruitment and in the informed consent procedures (e.g., publish the informed consent documentation as appendix, see also item X26), as this information may have an effect on user self-selection, user expectation and may also bias results.

	1	2	3	4	5	
subitem not at all important	○	○	○	○	◉	essential

Does your paper address subitem 4a-iii?

Copy and paste relevant sections from the manuscript (include quotes in quotation marks "like this" to indicate direct quotes from your manuscript), or elaborate on this item by providing additional information not in the ms, or briefly explain why the item is not applicable/relevant for your study

"We recruited participants through advertisements placed around the Cornell University campus. In the advertisement, we invited participants to test a new mobile application to help them stay on track for physical activity and food intake. Recruitment was restricted to participants who owned an Android smartphone and had interest in fitness. Prior to the study, the investigators arranged face-to-face meetings with the participants and acquired their informed consent. Participants also completed a brief survey to provide demographic data and information about their prior experience with mobile technologies and weight loss/fitness applications. All participants attended a training session, where they installed MyBehavior on their primary smartphone and received basic instructions, including how to enter their gender, height, and weight and set up a weekly weight goal (i.e., lose weight, maintain weight, or gain weight)."

4b) Settings and locations where the data were collected

Does your paper address CONSORT subitem 4b? *

Copy and paste relevant sections from the manuscript (include quotes in quotation marks "like this" to indicate direct quotes from your manuscript), or elaborate on this item by providing additional information not in the ms, or briefly explain why the item is not applicable/relevant for your study

"We recruited seventeen participants through advertisements placed around the Cornell University campus."

4b-i) Report if outcomes were (self-)assessed through online questionnaires

Clearly report if outcomes were (self-)assessed through online questionnaires (as common in web-based trials) or otherwise.

	1	2	3	4	5	
subitem not at all important	○	○	○	○	●	essential

Does your paper address subitem 4b-i? *

Copy and paste relevant sections from the manuscript (include quotes in quotation marks "like this" to indicate direct quotes from your manuscript), or elaborate on this item by providing additional information not in the ms, or briefly explain why the item is not applicable/relevant for your study

"First, we use a suggestion rating survey to evaluate user intentions to follow the suggestions. Participants completed this survey after the 3-week study concluded. Participants rated the suggestions by indicating on a 1-5 scale on whether they would be willing and able to do the recommended action on an average day (where 5=Strongly Agrees that he/she can follow the suggestion; 1=Strongly Disagrees). Each participant rated 25 suggestions providing 425 ratings. Each participant rated 15 of their own personal suggestions and 10 generic prescriptive suggestions. In addition, we quantitatively measure behavior change for all participants using logs of daily physical activity and dietary intakes.

The daily diary and the in-depth semi-structured interviews measured participant feedback regarding the suggestions. For the daily diaries, we queried (1) whether they looked at MyBehavior's suggestions, (2) whether they made or wanted to make any changes after seeing the suggestions. The semi-structured interviews covered users' general overall experience with MyBehavior and quality of the suggestions. Specifically, we inquired about awareness, behavior change, and if any software improvement they would like to see. In addition, in the interview, we asked clarifying questions that explain quantitative results observed from the data."

4b-ii) Report how institutional affiliations are displayed

Report how institutional affiliations are displayed to potential participants [on ehealth media], as affiliations with prestigious hospitals or universities may affect volunteer rates, use, and reactions with regards to an intervention.(Not a required item – describe only if this may bias results)

	1	2	3	4	5	
subitem not at all important	○	○	○	○	○	essential

Does your paper address subitem 4b-ii?

Copy and paste relevant sections from the manuscript (include quotes in quotation marks "like this" to indicate direct quotes from your manuscript), or elaborate on this item by providing additional information not in the ms, or briefly explain why the item is not applicable/relevant for your study

5) The interventions for each group with sufficient details to allow replication, including how and when

they were actually administered

5-i) Mention names, credential, affiliations of the developers, sponsors, and owners

Mention names, credential, affiliations of the developers, sponsors, and owners [6] (if authors/evaluators are owners or developer of the software, this needs to be declared in a “Conflict of interest” section or mentioned elsewhere in the manuscript).

	1	2	3	4	5	
subitem not at all important	○	○	○	○	●	essential

Does your paper address subitem 5-i?

Copy and paste relevant sections from the manuscript (include quotes in quotation marks "like this" to indicate direct quotes from your manuscript), or elaborate on this item by providing additional information not in the ms, or briefly explain why the item is not applicable/relevant for your study

"The team that supervised the trial included the builders of MyBehavior application and authors of this paper."

5-ii) Describe the history/development process

Describe the history/development process of the application and previous formative evaluations (e.g., focus groups, usability testing), as these will have an impact on adoption/use rates and help with interpreting results.

	1	2	3	4	5	
subitem not at all important	○	○	○	○	●	essential

Does your paper address subitem 5-ii?

Copy and paste relevant sections from the manuscript (include quotes in quotation marks "like this" to indicate direct quotes from your manuscript), or elaborate on this item by providing additional information not in the ms, or briefly explain why the item is not applicable/relevant for your study

Not applicable. We are describing the first version.

5-iii) Revisions and updating

Revisions and updating. Clearly mention the date and/or version number of the application/intervention (and comparator, if applicable) evaluated, or describe whether the intervention underwent major changes during the evaluation process, or whether the development and/or content was “frozen” during the trial. Describe dynamic components such as news feeds or changing content which may have an impact on the replicability of the intervention (for unexpected events see item 3b).

	1	2	3	4	5	
subitem not at all important	○	○	○	○	●	essential

Does your paper address subitem 5-iii?

Copy and paste relevant sections from the manuscript (include quotes in quotation marks "like this" to indicate direct quotes from your manuscript), or elaborate on this item by providing additional information not in the ms, or briefly explain why the item is not applicable/relevant for your study

not applicable.

5-iv) Quality assurance methods

Provide information on quality assurance methods to ensure accuracy and quality of information provided [1], if applicable.

	1	2	3	4	5	
subitem not at all important	○	○	○	○	●	essential

Does your paper address subitem 5-iv?

Copy and paste relevant sections from the manuscript (include quotes in quotation marks "like this" to indicate direct quotes from your manuscript), or elaborate on this item by providing additional information not in the ms, or briefly explain why the item is not applicable/relevant for your study

not applicable

5-v) Ensure replicability by publishing the source code, and/or providing screenshots/screen-capture video, and/or providing flowcharts of the algorithms used

Ensure replicability by publishing the source code, and/or providing screenshots/screen-capture video, and/or providing flowcharts of the algorithms used. Replicability (i.e., other researchers should in principle be able to replicate the study) is a hallmark of scientific reporting.

	1	2	3	4	5	
subitem not at all important	○	○	○	○	◉	essential

Does your paper address subitem 5-v?

Copy and paste relevant sections from the manuscript (include quotes in quotation marks "like this" to indicate direct quotes from your manuscript), or elaborate on this item by providing additional information not in the ms, or briefly explain why the item is not applicable/relevant for your study

We are not publishing the code right now since we would like to do more study on the app and publish more papers. Also, we will comment the source code and make it available soon. A video showing the app's functionalities is attached in the multi-media appendix of this paper.

5-vi) Digital preservation

Digital preservation: Provide the URL of the application, but as the intervention is likely to change or disappear over the course of the years; also make sure the intervention is archived (Internet Archive, webcitation.org, and/or publishing the source code or screenshots/videos alongside the article). As pages behind login screens cannot be archived, consider creating demo pages which are accessible without login.

	1	2	3	4	5	
subitem not at all important	○	○	○	○	◉	essential

Does your paper address subitem 5-vi?

Copy and paste relevant sections from the manuscript (include quotes in quotation marks "like this" to indicate direct quotes from your manuscript), or elaborate on this item by providing additional information not in the ms, or briefly explain why the item is not applicable/relevant for your study

not applicable

5-vii) Access

Access: Describe how participants accessed the application, in what setting/context, if they had to pay (or were paid) or not, whether they had to be a member of specific group. If known, describe how participants obtained "access to the platform and Internet" [1]. To ensure access for editors/reviewers/readers, consider to provide a "backdoor" login account or demo mode for reviewers/readers to explore the application (also important for archiving purposes, see vi).

	1	2	3	4	5	
subitem not at all important	○	○	○	○	◉	essential

Does your paper address subitem 5-vii? *

Copy and paste relevant sections from the manuscript (include quotes in quotation marks "like this" to indicate direct quotes from your manuscript), or elaborate on this item by providing additional information not in the ms, or briefly explain why the item is not applicable/relevant for your study

Participant didn't pay to access the app. Also, we will not publish the data collected during the study at this moment. The data has many privacy sensitive information, namely location. Our IRB approval doesn't give provisions to disclose the data collected outside of our research group at this moment.

Finally, this app is not freely available to the public now.

5-viii) Mode of delivery, features/functionalities/components of the intervention and comparator, and the theoretical framework

Describe mode of delivery, features/functionalities/components of the intervention and comparator, and the theoretical framework [6] used to design them (instructional strategy [1], behaviour change techniques, persuasive features, etc., see e.g., [7, 8] for terminology). This includes an in-depth description of the content (including where it is coming from and who developed it) [1]," whether [and how] it is tailored to individual circumstances and allows users to track their progress and receive feedback" [6]. This also includes a description of communication delivery channels and – if computer-mediated communication is a component – whether communication was synchronous or asynchronous [6]. It also includes information on presentation strategies [1], including page design principles, average amount of text on pages, presence of hyperlinks to other resources, etc. [1].

	1	2	3	4	5	
subitem not at all important	○	○	○	○	◉	essential

Does your paper address subitem 5-viii? *

Copy and paste relevant sections from the manuscript (include quotes in quotation marks "like this" to indicate direct quotes from your manuscript), or elaborate on this item by providing additional information not in the ms, or briefly explain why the item is not applicable/relevant for your study

"MyBehavior uses an exploit-explore strategy to automatically generate suggestions based on users' past physical activities and food intake. This suggestions generating strategy is grounded in contemporary behavioral science theories: (1) learning theory [19], (2) social cognitive theory [23], and (3) the Fogg behavior model (FBM) [26]. Behavior analysis applies learning theory first to assess whether a person has the skills needed to perform a behavior [19]. If so, the next step is to increase or decrease the target behavior's frequency by harnessing its antecedents (its setting and cues) and consequences (reinforcement). For example, if a health suggestion asks a user to swim but the user can't swim (i.e., he never acquired the skills), the user will not follow the suggestion. On the other hand, if a person has performed a behavior before, even if rarely, the skills can be assumed present. The Fogg behavior model applies theoretical principles to technology design by creating tools to prompt low effort actions that can be triggered even when motivation is low [26]. Thus, MyBehavior suggests (cues or triggers) a frequent behavior (e.g., a particular walk) that the person often does in a particular life context. This small, low effort change simply increases the frequency of a behavior that the person already does. Sometimes, instead, MyBehavior suggests an infrequent behavior (e.g., bike ride) that would burn more calories and that the person has shown he/she can do, but does only rarely. Social cognitive theory [23], the most widely used behavioral theory, suggests that in order to voluntarily initiate an action, a person needs a sense of self-efficacy or confidence that he/she will be able to perform it. The more frequently the person can be triggered to ride a bike repeatedly in a certain context where bikes are accessible, the more self-efficacy increases, the less effortful the behavior becomes, and the more likely that bike riding becomes a habit.

MyBehavior exploits the frequency principle by suggesting activities that users perform repeatedly. In addition, the algorithm favors actions that are not only frequent but also result in higher calorie expenditure. For example, short one-minute walks inside the office though very frequent, are likely to be superseded in the suggestion generation engine by a less frequent but higher calorie burning gym class. On the other hand, if the person rarely visits the gym but walks 30 minutes to work several times a week, the recommender engine will rank the walk higher than the gym since the aggregate calorie loss (frequency x calorie burnt each instance) is higher.

For stationary activities, the recommender engine suggests small changes, such as walking three minutes for every hour-spent stationary. Figure 4d shows a prioritization order of MyBehavior suggestions where simply adding 3-minute walks to the user's hour long stationary episodes burns more calories compared to rarely occurring gym visits.

Exploit suggestions generated solely from users' frequent past behavior may not generate sufficient energy expenditure to cause weight loss. Consequently, MyBehavior periodically suggests higher calorie burning activities to entice the user to try out and adopt. Explore suggestions target infrequent high calorie burning behaviors that the user can turn into a more regular activity. Future behavior is only imperfectly predicted by past behavior, and it could be the case that users will increase infrequent activities if suggested. Hence, if a user walks regularly near her office but sometimes goes to the gym or takes a long walk home, MyBehavior exploits this knowledge by suggesting walking near office most of the time and sometimes [illegible]

suggesting a gym visit or a long walk. If the new suggestion sticks and user starts going to the gym regularly as a result, then MyBehavior learns to target the gym as an exploit suggestion rather than an explore suggestion.

When generating food suggestions, a separate set of suggestions is created based on the exploit-explore strategy. First, MyBehavior distinguishes between meals and snacks. Then it takes into account both intake frequency and calories similar to the physical activity suggestions. Thus, a user's frequent healthy low calorie meals are exploited and are encouraged to be continued. During exploration, a random selection of infrequent low calorie meals/snacks from the past is suggested. Here the expectation is that users will uptake some of these infrequent meals and make them frequent in the future.

At the start of every day, MyBehavior generates ten food and ten activity suggestions. Of these, 90% are from the users' most frequent activities (i.e, exploit) and 10% are from the users' infrequent behaviors (e.g., explore). This split of 90% exploit and 10% explore was heuristically chosen based on previous literature [20]. This kind of exploit-explore strategy, well grounded in artificial intelligence research, falls under a wider decision making framework called Multi-armed bandit (MAB) [20]. MAB models have been well-studied for modeling dynamic systems where situations can change over time. In our case, user behavior isn't fixed and can change over time under MyBehavior's influence (Figure 5a and 5d). The exploit and explore strategy models this dynamic nature of human behavior effectively. MyBehavior exploits the most common user behaviors that promote energy balance to produce short-term health gain. To target long term health, it occasionally explores infrequent higher energy expending behaviors to discover actions that the user might repeats in the future, leading to sustained energy balance that could boost weight loss.

Figure 4 shows different generated suggestions that encourage the user to either continue positive activities (i.e., low calorie foods, walking, or exercise), make small changes in some situations (i.e., stationary activities) (Figure 4a) and to avoid negative activities (i.e., frequent large meals) (Figure 4b). Figure 4a and 4c are suggestions for two different users and Figure 4a and 4d are suggestions for the same user that change overtime."

" We provided MyBehavior's personalized context-sensitive suggestions to the experimental group while the control group received generic prescriptive recommendations generated from a pool of 42 suggestions for healthy living, such as "walk for 30 minutes" and "eat fish for dinner." A certified fitness professional created these generic suggestions after following National Institute of Health resources [30,31]. An external nutrition counselor also reviewed the suggestions to ensure that they were both healthy and achievable. For the following two weeks, participants continued to log behaviors, received their respective suggestions in their smartphones. During the entire study period, we asked participants to complete web-based daily diaries to better understand their

5-ix) Describe use parameters

Describe use parameters (e.g., intended "doses" and optimal timing for use). Clarify what instructions

or recommendations were given to the user, e.g., regarding timing, frequency, heaviness of use, if any, or was the intervention used ad libitum.

	1	2	3	4	5	
subitem not at all important	○	○	○	○	◉	essential

Does your paper address subitem 5-ix?

Copy and paste relevant sections from the manuscript (include quotes in quotation marks "like this" to indicate direct quotes from your manuscript), or elaborate on this item by providing additional information not in the ms, or briefly explain why the item is not applicable/relevant for your study

Automated and control condition suggestions were always available for participants to see whenever they want.

5-x) Clarify the level of human involvement

Clarify the level of human involvement (care providers or health professionals, also technical assistance) in the e-intervention or as co-intervention (detail number and expertise of professionals involved, if any, as well as “type of assistance offered, the timing and frequency of the support, how it is initiated, and the medium by which the assistance is delivered”. It may be necessary to distinguish between the level of human involvement required for the trial, and the level of human involvement required for a routine application outside of a RCT setting (discuss under item 21 – generalizability).

	1	2	3	4	5	
subitem not at all important	○	○	○	○	◉	essential

Does your paper address subitem 5-x?

Copy and paste relevant sections from the manuscript (include quotes in quotation marks "like this" to indicate direct quotes from your manuscript), or elaborate on this item by providing additional information not in the ms, or briefly explain why the item is not applicable/relevant for your study

No assistance was provided to use the app. The app was fully automated and the participants checked the app at their own discretion.

5-xi) Report any prompts/reminders used

Report any prompts/reminders used: Clarify if there were prompts (letters, emails, phone calls, SMS) to use the application, what triggered them, frequency etc. It may be necessary to distinguish between the level of prompts/reminders required for the trial, and the level of prompts/reminders for a routine application outside of a RCT setting (discuss under item 21 – generalizability).

1 2 3 4 5

subitem not at all important ◯ ◯ ◯ ◯ ◉ essential

Does your paper address subitem 5-xi? *

Copy and paste relevant sections from the manuscript (include quotes in quotation marks "like this" to indicate direct quotes from your manuscript), or elaborate on this item by providing additional information not in the ms, or briefly explain why the item is not applicable/relevant for your study

No prompts or reminders were used.

5-xii) Describe any co-interventions (incl. training/support)

Describe any co-interventions (incl. training/support): Clearly state any interventions that are provided in addition to the targeted eHealth intervention, as ehealth intervention may not be designed as stand-alone intervention. This includes training sessions and support [1]. It may be necessary to distinguish between the level of training required for the trial, and the level of training for a routine application outside of a RCT setting (discuss under item 21 – generalizability.

1 2 3 4 5

subitem not at all important ◯ ◯ ◯ ◯ ◉ essential

Does your paper address subitem 5-xii? *

Copy and paste relevant sections from the manuscript (include quotes in quotation marks "like this" to indicate direct quotes from your manuscript), or elaborate on this item by providing additional information not in the ms, or briefly explain why the item is not applicable/relevant for your study

Not applicable.

6a) Completely defined pre-specified primary and secondary outcome measures, including how and when they were assessed

Does your paper address CONSORT subitem 6a? *

Copy and paste relevant sections from the manuscript (include quotes in quotation marks "like this" to indicate direct quotes from your manuscript), or elaborate on this item by providing additional information not in the ms, or briefly explain why the item is not applicable/relevant for your study

"First, we use a suggestion rating survey to evaluate user intentions to follow the suggestions. Participants completed this survey after the 3-week study concluded. Participants rated the suggestions by indicating on a 1-5 scale on whether they would be willing and able to do the recommended action on an average day (where 5=Strongly Agrees that he/she can follow the suggestion; 1=Strongly Disagrees). Each participant rated 25 suggestions providing 425 ratings. Each participant rated 15 of their own personal suggestions and 10 generic prescriptive suggestions. In addition, we quantitatively measure behavior change for all participants using logs of daily physical activity and dietary intakes.

The daily diary and the in-depth semi-structured interviews measured participant feedback regarding the suggestions. For the daily diaries, we queried (1) whether they looked at MyBehavior's suggestions, (2) whether they made or wanted to make any changes after seeing the suggestions. The semi-structured interviews covered users' general overall experience with MyBehavior and quality of the suggestions. Specifically, we inquired about awareness, behavior change, and if any software improvement they would like to see. In addition, in the interview, we asked clarifying questions that explain quantitative results observed from the data."

6a-i) Online questionnaires: describe if they were validated for online use and apply CHERRIES items to describe how the questionnaires were designed/deployed

If outcomes were obtained through online questionnaires, describe if they were validated for online use and apply CHERRIES items to describe how the questionnaires were designed/deployed [9].

	1	2	3	4	5	
subitem not at all important	○	○	○	○	○	essential

Does your paper address subitem 6a-i?

Copy and paste relevant sections from manuscript text

6a-ii) Describe whether and how "use" (including intensity of use/dosage) was defined/measured/monitored

Describe whether and how "use" (including intensity of use/dosage) was defined/measured/monitored (logins, logfile analysis, etc.). Use/adoption metrics are important process outcomes that should be reported in any ehealth trial.

1 2 3 4 5

subitem not at all important ○ ○ ○ ○ ● essential

Does your paper address subitem 6a-ii?

Copy and paste relevant sections from manuscript text

"17 participants completed the three-week study, yielding almost 2.1 million recorded physical activity instances, amounting to more than 8000 hours of physical activities. During the same period, participants labeled nearly 850 images of food with annotations."

6a-iii) Describe whether, how, and when qualitative feedback from participants was obtained

Describe whether, how, and when qualitative feedback from participants was obtained (e.g., through emails, feedback forms, interviews, focus groups).

1 2 3 4 5

subitem not at all important ○ ○ ○ ○ ● essential

Does your paper address subitem 6a-iii?

Copy and paste relevant sections from manuscript text

"The daily diary and the in-depth semi-structured interviews measured participant feedback regarding the suggestions. For the daily diaries, we queried (1) whether they looked at MyBehavior's suggestions, (2) whether they made or wanted to make any changes after seeing the suggestions. The semi-structured interviews covered users' general overall experience with MyBehavior and quality of the suggestions. Specifically, we inquired about awareness, behavior change, and if any software improvement they would like to see. In addition, in the interview, we asked clarifying questions that explain quantitative results observed from the data."

"For the following two weeks, participants continued to log behaviors, received their respective suggestions in their smartphones. During the entire study period, we asked participants to complete web-based daily diaries to better understand their experience in following the suggestions provided. At the conclusion of the three week period, all participants were asked to complete a brief survey about the suggestions provided and were interviewed again face-to-face about their experience with the application."

6b) Any changes to trial outcomes after the trial commenced, with reasons

Does your paper address CONSORT subitem 6b? *

Copy and paste relevant sections from the manuscript (include quotes in quotation marks "like this" to indicate direct quotes from your manuscript), or elaborate on this item by providing additional information not in the ms, or briefly explain why the item is not applicable/relevant for your study

No changes were made.

7a) How sample size was determined

NPT: When applicable, details of whether and how the clustering by care provides or centers was addressed

7a-i) Describe whether and how expected attrition was taken into account when calculating the sample size

Describe whether and how expected attrition was taken into account when calculating the sample size.

	1	2	3	4	5	
subitem not at all important	○	○	○	○	●	essential

Does your paper address subitem 7a-i?

Copy and paste relevant sections from manuscript title (include quotes in quotation marks "like this" to indicate direct quotes from your manuscript), or elaborate on this item by providing additional information not in the ms, or briefly explain why the item is not applicable/relevant for your study

"We recruited 18 participants, 17 of which completed the study: 13 students and 4 professionals, 8 females and 9 males, all between the ages of 18 and 49 (μ=28.3; σ = 6.96; q25 = 22; q50 = 26.3; q75 = 36). Our sample size was determined based on earlier literature [63, 64, 67] suggesting that small studies ($n \geq 4$) are more suitable to test early feasibility of novel behavior change technologies like MyBehavior."

7b) When applicable, explanation of any interim analyses and stopping guidelines

Does your paper address CONSORT subitem 7b? *

Copy and paste relevant sections from the manuscript (include quotes in quotation marks "like this" to indicate direct quotes from your manuscript), or elaborate on this item by providing additional information not in the ms, or briefly explain why the item is not applicable/relevant for your study

This is not applicable

8a) Method used to generate the random allocation sequence

NPT: When applicable, how care providers were allocated to each trial group

Does your paper address CONSORT subitem 8a? *

Copy and paste relevant sections from the manuscript (include quotes in quotation marks "like this" to indicate direct quotes from your manuscript), or elaborate on this item by providing additional information not in the ms, or briefly explain why the item is not applicable/relevant for your study

"We randomize participants into the experimental (n=9) and control groups (n=8)."

We used a random number generator or table without blocking or stratification.

8b) Type of randomisation; details of any restriction (such as blocking and block size)

Does your paper address CONSORT subitem 8b? *

Copy and paste relevant sections from the manuscript (include quotes in quotation marks "like this" to indicate direct quotes from your manuscript), or elaborate on this item by providing additional information not in the ms, or briefly explain why the item is not applicable/relevant for your study

We used a random number generator or table without blocking or stratification.

9) Mechanism used to implement the random allocation sequence (such as sequentially numbered containers), describing any steps taken to conceal the sequence until interventions were assigned

Does your paper address CONSORT subitem 9? *

Copy and paste relevant sections from the manuscript (include quotes in quotation marks "like this" to indicate direct quotes from your manuscript), or elaborate on this item by providing additional information not in the ms, or briefly explain why the item is not applicable/relevant for your study

We used a random number generator or table without blocking or stratification.

10) Who generated the random allocation sequence, who enrolled participants, and who assigned participants to interventions

Does your paper address CONSORT subitem 10? *

Copy and paste relevant sections from the manuscript (include quotes in quotation marks "like this" to indicate direct quotes from your manuscript), or elaborate on this item by providing additional information not in the ms, or briefly explain why the item is not applicable/relevant for your study

The investigators "randomize participants into the experimental (n=9) and control groups (n=8)"

"The study investigators recruited seventeen participants through advertisements placed around the Cornell University campus. Recruitment was restricted to participants who owned a smartphone and had interest in fitness"

The PIs and the authors in the study performed and enrolled the participants.

11a) If done, who was blinded after assignment to interventions (for example, participants, care providers, those assessing outcomes) and how

NPT: Whether or not administering co-interventions were blinded to group assignment

11a-i) Specify who was blinded, and who wasn't

Specify who was blinded, and who wasn't. Usually, in web-based trials it is not possible to blind the participants [1, 3] (this should be clearly acknowledged), but it may be possible to blind outcome assessors, those doing data analysis or those administering co-interventions (if any).

	1	2	3	4	5	
subitem not at all important	○	○	○	○	●	essential

Does your paper address subitem 11a-i? *

Copy and paste relevant sections from the manuscript (include quotes in quotation marks "like this" to indicate direct quotes from your manuscript), or elaborate on this item by providing additional information not in the ms, or briefly explain why the item is not applicable/relevant for your study

"This study was single blind. i.e., the study subjects didn't know their experiment conditions, but the experimenters had full knowledge about subjects' experiment conditions."

These two conditions were not described in the consent form. The consent form described the following "we invite participants to test a new mobile application to help them stay on track for physical activity and food intake"

11a-ii) Discuss e.g., whether participants knew which intervention was the "intervention of interest" and which one was the "comparator"

Informed consent procedures (4a-ii) can create biases and certain expectations - discuss e.g., whether participants knew which intervention was the "intervention of interest" and which one was the "comparator".

	1	2	3	4	5	
subitem not at all important	○	○	○	○	◉	essential

Does your paper address subitem 11a-ii?

Copy and paste relevant sections from the manuscript (include quotes in quotation marks "like this" to indicate direct quotes from your manuscript), or elaborate on this item by providing additional information not in the ms, or briefly explain why the item is not applicable/relevant for your study

"This study was single blind. i.e., the study subjects didn't know their experiment conditions, but the experimenters had full knowledge about subjects' experiment conditions."

11b) If relevant, description of the similarity of interventions

(this item is usually not relevant for ehealth trials as it refers to similarity of a placebo or sham intervention to a active medication/intervention)

Does your paper address CONSORT subitem 11b? *

Copy and paste relevant sections from the manuscript (include quotes in quotation marks "like this" to indicate direct quotes from your manuscript), or elaborate on this item by providing additional information not in the ms, or briefly explain why the item is not applicable/relevant for your study

Our interventions are similar in that they both provided suggestions, one was general and one was contextualized and personalized.

12a) Statistical methods used to compare groups for primary and secondary outcomes

NPT: When applicable, details of whether and how the clustering by care providers or centers was addressed

Does your paper address CONSORT subitem 12a? *

Copy and paste relevant sections from the manuscript (include quotes in quotation marks "like this" to indicate direct quotes from your manuscript), or elaborate on this item by providing additional information not in the ms, or briefly explain why the item is not applicable/relevant for your study

"We used a fisher exact test to measure the number of positive changes as an effect of MyBehavior. Because of small sample size, the fisher exact test is used instead of the chi-square test for independence. We used two-sample independent student t-test to measure statistical significance for total walk lengths and total food calories consumed per day."

12a-i) Imputation techniques to deal with attrition / missing values

Imputation techniques to deal with attrition / missing values: Not all participants will use the intervention/comparator as intended and attrition is typically high in ehealth trials. Specify how participants who did not use the application or dropped out from the trial were treated in the statistical analysis (a complete case analysis is strongly discouraged, and simple imputation techniques such as LOCF may also be problematic [4]).

	1	2	3	4	5	
subitem not at all important	○	○	○	○	●	essential

Does your paper address subitem 12a-i? *

Copy and paste relevant sections from the manuscript (include quotes in quotation marks "like this" to indicate direct quotes from your manuscript), or elaborate on this item by providing additional information not in the ms, or briefly explain why the item is not applicable/relevant for your study

Not applicable. We didn't fill missing values.

12b) Methods for additional analyses, such as subgroup analyses and adjusted analyses

Does your paper address CONSORT subitem 12b? *

Copy and paste relevant sections from the manuscript (include quotes in quotation marks "like this" to indicate direct quotes from your manuscript), or elaborate on this item by providing additional information not in the ms, or briefly explain why the item is not applicable/relevant for your study

Not applicable.

X26) REB/IRB Approval and Ethical Considerations [recommended as subheading under "Methods"] (not a CONSORT item)

X26-i) Comment on ethics committee approval

	1	2	3	4	5	
subitem not at all important	○	○	○	○	●	essential

Does your paper address subitem X26-i?

Copy and paste relevant sections from the manuscript (include quotes in quotation marks "like this" to indicate direct quotes from your manuscript), or elaborate on this item by providing additional information not in the ms, or briefly explain why the item is not applicable/relevant for your study

"This study was approved by Cornell University Institutional Review Board (Protocol #: 1302003617)"

x26-ii) Outline informed consent procedures

Outline informed consent procedures e.g., if consent was obtained offline or online (how? Checkbox, etc.?), and what information was provided (see 4a-ii). See [6] for some items to be included in informed consent documents.

	1	2	3	4	5	
subitem not at all important	○	○	○	○	●	essential

Does your paper address subitem X26-ii?

Copy and paste relevant sections from the manuscript (include quotes in quotation marks "like this" to indicate direct quotes from your manuscript), or elaborate on this item by providing additional information not in the ms, or briefly explain why the item is not applicable/relevant for your study

"Prior to the study, the investigators arranged face-to-face meetings with the participants and acquired their informed consent."

X26-iii) Safety and security procedures

Safety and security procedures, incl. privacy considerations, and any steps taken to reduce the likelihood or detection of harm (e.g., education and training, availability of a hotline)

	1	2	3	4	5	
subitem not at all important	○	○	○	○	◉	essential

Does your paper address subitem X26-iii?

Copy and paste relevant sections from the manuscript (include quotes in quotation marks "like this" to indicate direct quotes from your manuscript), or elaborate on this item by providing additional information not in the ms, or briefly explain why the item is not applicable/relevant for your study

"This study was approved by Cornell University Institutional Review Board (Protocol #: 1302003617) "

RESULTS

13a) For each group, the numbers of participants who were randomly assigned, received intended treatment, and were analysed for the primary outcome

NPT: The number of care providers or centers performing the intervention in each group and the number of patients treated by each care provider in each center

Does your paper address CONSORT subitem 13a? *

Copy and paste relevant sections from the manuscript (include quotes in quotation marks "like this" to indicate direct quotes from your manuscript), or elaborate on this item by providing additional information not in the ms, or briefly explain why the item is not applicable/relevant for your study

"We recruited 18 participants, 17 of which completed the study"

"Investigators randomly assigned participants to receive either MyBehavior's personalized suggestions (n=9) or non-personalized suggestions, created by professionals (n=8), from a smartphone application over 3 weeks."

These 17 participants were analyzed for result.

13b) For each group, losses and exclusions after randomisation, together with reasons

Does your paper address CONSORT subitem 13b? (NOTE: Preferably, this is shown in a CONSORT flow diagram) *

Copy and paste relevant sections from the manuscript (include quotes in quotation marks "like this" to indicate direct quotes from your manuscript), or elaborate on this item by providing additional information not in the ms, or briefly explain why the item is not applicable/relevant for your study

No losses or exclusions happened after randomization.

13b-i) Attrition diagram

Strongly recommended: An attrition diagram (e.g., proportion of participants still logging in or using the intervention/comparator in each group plotted over time, similar to a survival curve) or other figures or tables demonstrating usage/dose/engagement.

	1	2	3	4	5	
subitem not at all important	○	○	○	○	●	essential

Does your paper address subitem 13b-i?

Copy and paste relevant sections from the manuscript or cite the figure number if applicable (include quotes in quotation marks "like this" to indicate direct quotes from your manuscript), or elaborate on this item by providing additional information not in the ms, or briefly explain why the item is not applicable/relevant for your study

"Figure 1: Flow of participants in the trial" shows the participant flow for the study. This diagram shows details on participants and their engagement in the study.

14a) Dates defining the periods of recruitment and follow-up

Does your paper address CONSORT subitem 14a? *

Copy and paste relevant sections from the manuscript (include quotes in quotation marks "like this" to indicate direct quotes from your manuscript), or elaborate on this item by providing additional information not in the ms, or briefly explain why the item is not applicable/relevant for your study

The study took place over three weeks from May 2013 to June 2103 in two different phases: a baseline phase and an experimental phase.

14a-i) Indicate if critical "secular events" fell into the study period

Indicate if critical "secular events" fell into the study period, e.g., significant changes in Internet resources available or "changes in computer hardware or Internet delivery resources"

	1	2	3	4	5	
subitem not at all important	○	○	○	○	●	essential

Does your paper address subitem 14a-i?

Copy and paste relevant sections from the manuscript (include quotes in quotation marks "like this" to indicate direct quotes from your manuscript), or elaborate on this item by providing additional information not in the ms, or briefly explain why the item is not applicable/relevant for your study

No such event was included.

14b) Why the trial ended or was stopped (early)

Does your paper address CONSORT subitem 14b? *

Copy and paste relevant sections from the manuscript (include quotes in quotation marks "like this" to indicate direct quotes from your manuscript), or elaborate on this item by providing additional information not in the ms, or briefly explain why the item is not applicable/relevant for your study

not applicable

15) A table showing baseline demographic and clinical characteristics for each group

NPT: When applicable, a description of care providers (case volume, qualification, expertise, etc.) and centers (volume) in each group

Does your paper address CONSORT subitem 15? *

Copy and paste relevant sections from the manuscript (include quotes in quotation marks "like this" to indicate direct quotes from your manuscript), or elaborate on this item by providing additional information not in the ms, or briefly explain why the item is not applicable/relevant for your study

We verbally describe the demographics in the description as follows:

"The 17 participants were 13 students and 4 professionals, 8 females and 9 males, all between the ages of 18 and 49 (μ=28.3; σ = 6.96; q25 = 22; q50 = 26.3; q75 = 36). The majority of participants were experienced smartphone users; nine participants had previous experience using a food diary, and 6 participants had previously kept an exercise log."

15-i) Report demographics associated with digital divide issues

In ehealth trials it is particularly important to report demographics associated with digital divide issues, such as age, education, gender, social-economic status, computer/Internet/ehealth literacy of the participants, if known.

	1	2	3	4	5	
subitem not at all important	○	○	○	○	●	essential

Does your paper address subitem 15-i? *

Copy and paste relevant sections from the manuscript (include quotes in quotation marks "like this" to indicate direct quotes from your manuscript), or elaborate on this item by providing additional information not in the ms, or briefly explain why the item is not applicable/relevant for your study

Recruitment was restricted to participants who owned an Android smartphone and had interest in fitness.

16) For each group, number of participants (denominator) included in each analysis and whether the analysis was by original assigned groups

16-i) Report multiple “denominators” and provide definitions

Report multiple “denominators” and provide definitions: Report N’s (and effect sizes) “across a range of study participation [and use] thresholds” [1], e.g., N exposed, N consented, N used more than x times, N used more than y weeks, N participants “used” the intervention/comparator at specific pre-defined time points of interest (in absolute and relative numbers per group). Always clearly define “use” of the intervention.

	1	2	3	4	5	
subitem not at all important	○	○	○	○	●	essential

Does your paper address subitem 16-i? *

Copy and paste relevant sections from the manuscript (include quotes in quotation marks "like this" to indicate direct quotes from your manuscript), or elaborate on this item by providing additional information not in the ms, or briefly explain why the item is not applicable/relevant for your study

All participants were included for physical activity analysis.

However, for dietary behavior change we excluded one participants for less data. Exact line from manuscript is "1 participant had insufficient data"

16-ii) Primary analysis should be intent-to-treat

Primary analysis should be intent-to-treat, secondary analyses could include comparing only “users”, with the appropriate caveats that this is no longer a randomized sample (see 18-i).

	1	2	3	4	5	
subitem not at all important	○	○	○	○	○	essential

Does your paper address subitem 16-ii?

Copy and paste relevant sections from the manuscript (include quotes in quotation marks "like this" to indicate direct quotes from your manuscript), or elaborate on this item by providing additional information not in the ms, or briefly explain why the item is not applicable/relevant for your study

17a) For each primary and secondary outcome, results for each group, and the estimated effect size and its precision (such as 95% confidence interval)

Does your paper address CONSORT subitem 17a? *

Copy and paste relevant sections from the manuscript (include quotes in quotation marks "like this" to indicate direct quotes from your manuscript), or elaborate on this item by providing additional information not in the ms, or briefly explain why the item is not applicable/relevant for your study

User acceptance of MyBehavior suggestions
"In the suggestion rating survey, the experimental group (μ= 3.4; σ = 1.2; q25 = 2.75; q50 = 3; q75 = 4) with MyBehavior suggestions expressed greater intention to follow personalized suggestions than the control group (μ= 2.5; σ = 1.6; q25 = 1; q50 = 2; q75 = 4) intended to follow the generic suggestions. A non-parametric Mann-Whitney U test [62] found that this difference to be statistically significant ($p < 0.001$, 95% CI [0, 1.001], effect size = 0.99).

Physical Activity
Figures 5a and 5b show the distribution (box plots) of walking lengths over time for the experiment and the control groups. For each week of the study, we compute these distributions for the different users. To ease interpretation, we joined the median per week with thick green or red lines for each user. A green line implies a positive change as discussed in the data analysis section. The red line indicates the reverse negative trend. We used log scale for walking length distribution since walking length distributions have heavy tails [46].

Figure 5: (a)-(b) respectively show the distribution of walking lengths for experiment and control group over the 3-week study. We joined the medians of distributions and showed the trend as a thick green or red line. Green and red respectively means a decreasing and increasing trend median food calorie intake.

For walking, 78% (7/9) participants in the experimental group (Figure 6c) showed positive trends, whereas 75% (6/8) participants in the control group exhibited negative trends (Figure 6d). A fisher exact test found this ratio in number of positive changes statistically significant [45] ($p = 0.05$). In addition, MyBehavior users walked an average of 10 minutes more per day in the experiment phase (i.e., from 1st to third week). However, we did not observe any change for the control group. Two-sample t-test found this difference in change of walking duration to be significant ($t(15)= 2.1$, $p=0.055$, 95% CI = [-0.23, 19.052], $d = 0.9$).

Qualitative data from daily diary and face-to-face interviews largely support this quantitative result. However, we also observed some important subtleties. First, experimental group described the activity suggestions to be actionable and relevant to their lives. On the other hand, control group participants appreciated that the generic suggestion reminded them of good habits. However, they often faced problems incorporating the suggestions into their daily lives .
"Those suggestions are quite good, which reminds me not to sit too long in one place. [A1, diary]"
"The exercise suggestions made me want to do some more activities and be less stationary. Seeing how long I have been stationary and the low frequency of activity made me want to make a change. [A5, diary]"
"Try to get up from my desk more often... added walk" notes to my calendar [A2, diary]"
"I did some walking where I normally walk. The app now shows I walked there 26 times. The app makes me feel that I can do it again since I have done the same walk many times. [A7, daily diary]"
"The suggestions encourage me to do/plan exercises for the near future.... It reminds me that some foods are better than others. [G1, diary]"
"They seem like good generic suggestions. The kind you would read...as tips in a health magazine or some such.. [G4, diary]"

Some MyBehavior users reported that even the non-frequent explore suggestions were actionable and expressed interest in acting on them. For instance, subject A7 said,
"I saw a walk to my nearest bus stand listed. Normally, I drive my car to go to my office. But looking at the extra walking I got while going to the bus stop makes me think about doing it often and making it a habit. [A7, daily diary]"

Results from interviews also revealed that participants at various stages of active lifestyle reacted to suggestions differentially [54]. For the experimental group, participants likely considering making changes expressed that they became more self-conscious about their behavior and they were eager to follow the suggested changes (e.g., starting to walk more near home, or continuing runs on treadmills). Comparatively, users likely maintaining an active lifestyle expressed that the suggestion reflected their current healthy behavior and considered them good reinforcements. Still these maintaining participants wanted to change their stationary behavior in the office with occasional small walks. For the control group, users reported frustration in the lack of adaptation of suggestions and increasingly became less interested in the app. Control group users maintaining an active lifestyle were unaffected by generic suggestions and continued their regular behavior across weeks. For example, G7 and G8 were maintaining control group participants and their behavior showed no negative trends in Figure 5b. Control group users who already didn't have a maintaining lifestyle gradually became less active or made poorer food choices after the initial excitement phase of the fitness study.

Finally, on a few occasions, MyBehavior suggestions were hard to follow or did not reflect user preferences. For example, one user reported in the interview that he used to play soccer with his friends but his friends recently moved to a new location. He could no longer play soccer, which MyBehavior was suggesting. In addition, often user preferred activities are not top MyBehavior suggestions. For instance, one user preferred to swim even though she did not do it

often. Finally, A8 (subject 8 in figure 5a with negative trends) could not follow MyBehavior suggestions since he was working for a deadline during the study.

Dietary Behavior
Figure 6a and 6b respectively shows the distribution (box plots) of meal calories for experiment and control group. For each week of the study, we compute these distributions for different users. Similar to walking behavior graphs, we joined medians across weeks to show positive or negative change for each user.

Figure 6: (a)-(b) respectively show the distribution of food calories for experiment and control group over the 3-week study. We joined the medians of distributions and showed the trend as a thick green or red line. Green and red respectively means a decreasing and increasing trend median food calorie intake.

For caloric intake, 77.8% (7/9) of participants in the experimental group showed positive trends (green lines in Fig. 6a), and 57.1% (4/7) of participants in the control group showed negative trends (red lines in Fig. 6b; 1 participant had insufficient data). However, a fisher exact test found this to be non-significant ($p = 0.15$). For control group participants, we also found their average per day median calorie to increase by 211 calories ($\mu = 211.7$; $\sigma = 263.07$; $q25 = -31.25$; $q50 = 187.5$; $q75 = 429.35$) from 1st week to 3rd week. Comparatively, the experimental group showed an average per day calorie decrease by nearly 100 calories ($\mu = -99.3$; $\sigma = 481.27$; $q25 = -527.83$; $q50 = -37.3$; $q75 = 87.5$) from 1st week to 3rd. This change was not significant in a two-sample t-test ($t(12)=1.3234$, $p=0.21$, 95% CI=[-201,822.96], $d = 0.72$).

In qualitative feedback, similar to physical activity suggestions, experimental group users found the suggestions to be more actionable and reported to make changes compared to control group users who found the suggestions to be hard to work on
"the pictures of my meals are very useful to keep track of what I've been eating in the past. People tend to forget about their habits, but pictures in this case are a nice way to bring your food history in front of your eyes. [A9, daily diary]"
"The suggestions remind me that some foods are better than others. [G1, diary]"
"It recommends me to eat stuff that I don't have at home [G4, diary]"
"These suggestions don't take into account my dietary restrictions. [G5, diary]"

Similar to activity explore suggestions, MyBehavior users often found the explore suggestions to be actionable,
"I just wanted to see what it was.... These ones [explore suggestions] seemed to pick up some "good" food habits. [A4, daily diary]"

Finally, users reported manual food logging to be time consuming in

17a-i) Presentation of process outcomes such as metrics of use and intensity of use

In addition to primary/secondary (clinical) outcomes, the presentation of process outcomes such as metrics of use and intensity of use (dose, exposure) and their operational definitions is critical. This does not only refer to metrics of attrition (13-b) (often a binary variable), but also to more continuous exposure metrics such as "average session length". These must be accompanied by a technical

description how a metric like a “session” is defined (e.g., timeout after idle time) [1] (report under item 6a).

	1	2	3	4	5	
subitem not at all important	○	○	○	○	●	essential

Does your paper address subitem 17a-i?

Copy and paste relevant sections from the manuscript (include quotes in quotation marks "like this" to indicate direct quotes from your manuscript), or elaborate on this item by providing additional information not in the ms, or briefly explain why the item is not applicable/relevant for your study

"17 participants completed the three-week study, yielding almost 2.1 million recorded physical activity instances, amounting to more than 8000 hours of physical activities. During the same period, participants labeled nearly 850 images of food with annotations."

17b) For binary outcomes, presentation of both absolute and relative effect sizes is recommended

Does your paper address CONSORT subitem 17b? *

Copy and paste relevant sections from the manuscript (include quotes in quotation marks "like this" to indicate direct quotes from your manuscript), or elaborate on this item by providing additional information not in the ms, or briefly explain why the item is not applicable/relevant for your study

not applicable.

18) Results of any other analyses performed, including subgroup analyses and adjusted analyses, distinguishing pre-specified from exploratory

Does your paper address CONSORT subitem 18? *

Copy and paste relevant sections from the manuscript (include quotes in quotation marks "like this" to indicate direct quotes from your manuscript), or elaborate on this item by providing additional information not in the ms, or briefly explain why the item is not applicable/relevant for your study

not applicable

18-i) Subgroup analysis of comparing only users

A subgroup analysis of comparing only users is not uncommon in ehealth trials, but if done, it must be stressed that this is a self-selected sample and no longer an unbiased sample from a randomized trial (see 16-iii).

	1	2	3	4	5	
subitem not at all important	◯	◯	◯	◯	◯	essential

Does your paper address subitem 18-i?

Copy and paste relevant sections from the manuscript (include quotes in quotation marks "like this" to indicate direct quotes from your manuscript), or elaborate on this item by providing additional information not in the ms, or briefly explain why the item is not applicable/relevant for your study

19) All important harms or unintended effects in each group

(for specific guidance see CONSORT for harms)

Does your paper address CONSORT subitem 19? *

Copy and paste relevant sections from the manuscript (include quotes in quotation marks "like this" to indicate direct quotes from your manuscript), or elaborate on this item by providing additional information not in the ms, or briefly explain why the item is not applicable/relevant for your study

"MyBehavior is a mobile phone application with a suggestion generation mechanism. So the risk of intended harms or unintended effects are minimal. In our study, we didn't encounter any unintended effect."

19-i) Include privacy breaches, technical problems

Include privacy breaches, technical problems. This does not only include physical “harm” to participants, but also incidents such as perceived or real privacy breaches [1], technical problems, and

other unexpected/unintended incidents. “Unintended effects” also includes unintended positive effects [2].

	1	2	3	4	5	
subitem not at all important	◯	◯	◯	◯	◯	essential

Does your paper address subitem 19-i?

Copy and paste relevant sections from the manuscript (include quotes in quotation marks "like this" to indicate direct quotes from your manuscript), or elaborate on this item by providing additional information not in the ms, or briefly explain why the item is not applicable/relevant for your study

19-ii) Include qualitative feedback from participants or observations from staff/researchers

Include qualitative feedback from participants or observations from staff/researchers, if available, on strengths and shortcomings of the application, especially if they point to unintended/unexpected effects or uses. This includes (if available) reasons for why people did or did not use the application as intended by the developers.

	1	2	3	4	5	
subitem not at all important	◯	◯	◯	◯	◯	essential

Does your paper address subitem 19-ii?

Copy and paste relevant sections from the manuscript (include quotes in quotation marks "like this" to indicate direct quotes from your manuscript), or elaborate on this item by providing additional information not in the ms, or briefly explain why the item is not applicable/relevant for your study

Qualitative measures:
"Qualitative data from daily diary and face-to-face interviews largely support the quantitative result. However, we also observed some important subtleties. First, experimental group described the activity suggestions to be actionable and relevant to their lives. On the other hand, control group participants appreciated that the generic suggestion reminded them of good habits. However, they often faced problems incorporating the suggestions into their daily lives .
“Those suggestions are quite good, which reminds me not to sit too long in one place. [A1, diary]”
“The exercise suggestions made me want to do some more activities and be less stationary. Seeing how long I have been stationary and the low frequency of activity made me want to make a change. [A5, diary]”
“Try to get up from my desk more often... added walk" notes to my calendar [A2, diary]”
“I did some walking where I normally walk. The app now shows I walked there 26 times. The app makes me feel that I can do it again since I have done the same walk many times. [A7, daily diary]”
“The suggestions encourage me to do/plan exercises for the near future.... It reminds me that some foods are better than others. [G1, diary]”

diary]
"They seem like good generic suggestions. The kind you would read...as tips in a health magazine or some such.. [G4, diary]"

Some MyBehavior users reported that even the non-frequent explore suggestions are actionable and expressed interest in acting on them. For instance, subject A7 said,
"I saw a walk to my nearest bus stand listed. Normally, I drive my car to go to my office. But looking at the extra walking I got while going to the bus stop makes me think about doing it often and making it a habit. [A7, daily diary]"

An important subtlety was how participants at different stages of active lifestyle reacted to suggestions [54]. For experiment group, participants likely considering making changes informed that they became more self-conscious about their behavior and they were eager to follow the suggested changes (e.g., starting to walk more near home, or continuing runs on treadmills). On the other hand, users likely maintaining an active lifestyle informed the suggestion to reflect their current healthy behavior and considered them as good reinforcements. Still these maintaining participants wanted to change their stationary behavior in office with occasional small walks. For the control group, users considering making changes informed to become more active in first week but later couldn't sustain the changes. These users reported frustration in the lack of adaptation in suggestions and increasingly become less interested in the app. In contrary, users maintaining an active lifestyle were unaffected by generic suggestions and continued their regular behavior across weeks. e.g., G7 and G8 were maintaining control group participants and their behavior showed no negative trends in Figure 6d.

Finally, on a few occasions, MyBehavior suggestions were hard to follow or didn't reflect user preferences. For example, one user informed in the interview that he used to play soccer with his friends but his friends recently moved to a new location. He could no longer play soccer, which MyBehavior was suggesting. In addition, often user preferred activities that are not top MyBehavior suggestions. For instance, one user preferred to swim even though she didn't do it often. Finally, A8 with MyBehavior suggestions (subject 8 in figure 6c with negative trends) couldn't follow MyBehavior suggestions since he was working for a deadline during the study."

"In qualitative feedback, similar to physical activity suggestions, experiment group users found the suggestions to be more actionable and reported to make changes. On the other hand, control group users found the suggestions to be hard to work on
"the pictures of my meals are very useful to keep track of what I've been eating in the past. People tend to forget about their habits, but pictures in this case are a nice way to bring your food history in front of your eyes. [A9, daily diary]"
"The suggestions remind me that some foods are better than others. [G1, diary]"
"It recommends me to eat stuff that I don't have at home [G4, diary]"
"These suggestions don't take into account my dietary restrictions. [G5, diary]"

Similar to activity explore suggestions, MyBehavior users often found the explore suggestions to be actionable,
"I just wanted to see what it was.... These ones [explore suggestions] seemed to pick up some "good" food habits. [A4, daily diary]"

Finally, users reported manual food logging to be time consuming. However, they also reported this manual process made them more

aware of their foods. Even, control group participants reported to make dietary changes without personalized suggestions. "

DISCUSSION

22) Interpretation consistent with results, balancing benefits and harms, and considering other relevant evidence

NPT: In addition, take into account the choice of the comparator, lack of or partial blinding, and unequal expertise of care providers or centers in each group

22-i) Restate study questions and summarize the answers suggested by the data, starting with primary outcomes and process outcomes (use)

Restate study questions and summarize the answers suggested by the data, starting with primary outcomes and process outcomes (use).

	1	2	3	4	5	
subitem not at all important	○	○	○	○	◉	essential

Does your paper address subitem 22-i? *

Copy and paste relevant sections from the manuscript (include quotes in quotation marks "like this" to indicate direct quotes from your manuscript), or elaborate on this item by providing additional information not in the ms, or briefly explain why the item is not applicable/relevant for your study

"To our knowledge, MyBehavior is the first system to automatically provide personalized suggestions that relates to users' lifestyle. In the quantitative results, MyBehavior users demonstrated superior behavior changes compared to the control. Qualitative measures from the face-to-face interviews and the daily diary confirmed that the suggestions indeed were perceived to be personalized to their lives. This concordance of superiority in both quantitative behavior change and qualitative user perception makes MyBehavior's automated health feedback approach potentially feasible and provides support for longitudinal studies and future investigations into automated personalization approaches.

Specifically, in our evaluation, users rated that they can follow MyBehavior personalized suggestions more than the control condition suggestions. Results also revealed a significant change in walking behaviors for MyBehavior users. In qualitative measures, users reported MyBehavior activity suggestions to be more actionable. Interestingly, although users qualitatively reported the dietary suggestions to be more actionable, dietary behavior changes were not found to be different between the groups. This finding could be due to the manual logging nature of food intake as sufficient for behavior change alone. The manual process of food logging might produce self-awareness and reflection. Indeed, past research demonstrates that the simple logging can improve one's food consumption behavior [66]. However, food logging is an arduous process and it is often hard to continue for an extended period. Thus, we need longer studies to determine if food logging along with suggestions could aid in sustained behavior change. Furthermore, we had a small sample in the study with inadequate statistical power. Thus, larger trials are necessary to further elucidate the effects of food logging and these types of suggestions on eating behavior."

22-ii) Highlight unanswered new questions, suggest future research

Highlight unanswered new questions, suggest future research.

	1	2	3	4	5	
subitem not at all important	○	○	○	○	●	essential

Does your paper address subitem 22-ii?

Copy and paste relevant sections from the manuscript (include quotes in quotation marks "like this" to indicate direct quotes from your manuscript), or elaborate on this item by providing additional information not in the ms, or briefly explain why the item is not applicable/relevant for your study

"Despite this promising direction, the automated data-driven personalization approach of MyBehavior brings its own challenges. Manual logging of food and exercise, in addition to automated logging, are necessary for proper functioning of MyBehavior. Qualitative interviews revealed that manual food and exercise logging were often burdensome. Future iterations could use crowdsourcing based semi-automated approaches to decrease burden of manual food journaling [16]. Finally, interviews also highlighted the importance of considering contextual changes in users' lives and preference. Thus, giving users control in deciding which suggestion they want to follow is required for well-accepted personalization [18]."

20) Trial limitations, addressing sources of potential bias, imprecision, and, if relevant, multiplicity of analyses

20-i) Typical limitations in ehealth trials

Typical limitations in ehealth trials: Participants in ehealth trials are rarely blinded. Ehealth trials often look at a multiplicity of outcomes, increasing risk for a Type I error. Discuss biases due to non-use of the intervention/usability issues, biases through informed consent procedures, unexpected events.

	1	2	3	4	5	
subitem not at all important	○	○	○	○	●	essential

Does your paper address subitem 20-i? *

Copy and paste relevant sections from the manuscript (include quotes in quotation marks "like this" to indicate direct quotes from your manuscript), or elaborate on this item by providing additional information not in the ms, or briefly explain why the item is not applicable/relevant for your study

"An important limitation of this study is the short-term and small-scale nature of the study, making conclusions difficult. However, this study helped us identify the potential efficacy of MyBehavior and pinpoint design improvements for future deployments. Indeed, Klasanja et al. [64] argue that such short-term studies with similar evaluation goals as our study are often more suitable for new and untested behavior change technologies like MyBehavior. Another limitation was that the non-personalized suggestions were sometimes too specific, e.g., “walking with a dog”. In the daily diaries, some users reported they could not follow this suggestion since they did not own a dog. While designing generic suggestions, we tried to find suggestions that most users could follow, without being overly generic. Despite this effort, there will always be exceptions where a suggestion does not fit one’s lifestyle."

21) Generalisability (external validity, applicability) of the trial findings

NPT: External validity of the trial findings according to the intervention, comparators, patients, and care providers or centers involved in the trial

21-i) Generalizability to other populations

Generalizability to other populations: In particular, discuss generalizability to a general Internet population, outside of a RCT setting, and general patient population, including applicability of the study results for other organizations

	1	2	3	4	5	
subitem not at all important	○	○	○	○	●	essential

Does your paper address subitem 21-i?

Copy and paste relevant sections from the manuscript (include quotes in quotation marks "like this" to indicate direct quotes from your manuscript), or elaborate on this item by providing additional information not in the ms, or briefly explain why the item is not applicable/relevant for your study

"An important limitation of this study is the short-term and small-scale nature of the study, making conclusions difficult. However, this study helped us identify the potential efficacy of MyBehavior and pinpoint design improvements for future deployments. Indeed, Klasanja et al. [64] argue that such short-term studies with similar evaluation goals as our study are often more suitable for new and untested behavior change technologies like MyBehavior."

21-ii) Discuss if there were elements in the RCT that would be different in a routine application

setting

Discuss if there were elements in the RCT that would be different in a routine application setting (e.g., prompts/reminders, more human involvement, training sessions or other co-interventions) and what impact the omission of these elements could have on use, adoption, or outcomes if the intervention is applied outside of a RCT setting.

	1	2	3	4	5	
subitem not at all important	◯	◯	◯	◯	◯	essential

Does your paper address subitem 21-ii?

Copy and paste relevant sections from the manuscript (include quotes in quotation marks "like this" to indicate direct quotes from your manuscript), or elaborate on this item by providing additional information not in the ms, or briefly explain why the item is not applicable/relevant for your study

OTHER INFORMATION

23) Registration number and name of trial registry

Does your paper address CONSORT subitem 23? *

Copy and paste relevant sections from the manuscript (include quotes in quotation marks "like this" to indicate direct quotes from your manuscript), or elaborate on this item by providing additional information not in the ms, or briefly explain why the item is not applicable/relevant for your study

clinicaltrials.gov

NCT02359981

24) Where the full trial protocol can be accessed, if available

Does your paper address CONSORT subitem 24? *

Cite a Multimedia Appendix, other reference, or copy and paste relevant sections from the manuscript (include quotes in quotation marks "like this" to indicate direct quotes from your manuscript), or elaborate on this item by providing additional information not in the ms, or briefly explain why the item is not applicable/relevant for your study

https://clinicaltrials.gov/ct2/show/NCT02359981

25) Sources of funding and other support (such as supply of drugs), role of funders

Does your paper address CONSORT subitem 25? *

Copy and paste relevant sections from the manuscript (include quotes in quotation marks "like this" to indicate direct quotes from your manuscript), or elaborate on this item by providing additional information not in the ms, or briefly explain why the item is not applicable/relevant for your study

This study if funded by Intel ISTC for pervasive computing and NSF #0845683

X27) Conflicts of Interest (not a CONSORT item)

X27-i) State the relation of the study team towards the system being evaluated

In addition to the usual declaration of interests (financial or otherwise), also state the relation of the study team towards the system being evaluated, i.e., state if the authors/evaluators are distinct from or identical with the developers/sponsors of the intervention.

	1	2	3	4	5	
subitem not at all important	○	○	○	○	●	essential

Does your paper address subitem X27-i?

Copy and paste relevant sections from the manuscript (include quotes in quotation marks "like this" to indicate direct quotes from your manuscript), or elaborate on this item by providing additional information not in the ms, or briefly explain why the item is not applicable/relevant for your study

"To evaluate the feasibility of MyBehavior, a small 3-week two-group randomized control trial was conducted. The team that supervised the trial included the builders of MyBehavior application and authors of this paper. This team recruited participants through advertisements placed around the Cornell University campus. In the advertisement, we invited participants to test a new mobile application to help them stay on track for physical activity and food intake."

About the CONSORT EHEALTH checklist

As a result of using this checklist, did you make changes in your manuscript? *

- (x) yes, major changes
- ( ) yes, minor changes
- ( ) no

What were the most important changes you made as a result of using this checklist?

We restructured the documents after filling out the consort from. Also, small details about conflict of interest are addressed in the paper after filling out consort form.

How much time did you spend on going through the checklist INCLUDING making changes in your manuscript *

10 hours

As a result of using this checklist, do you think your manuscript has improved? *

- (x) yes
- ( ) no
- ( ) Other:

Would you like to become involved in the CONSORT EHEALTH group?

This would involve for example becoming involved in participating in a workshop and writing an "Explanation and Elaboration" document

- ( ) yes
- (x) no
- ( ) Other:

Any other comments or questions on CONSORT EHEALTH

STOP - Save this form as PDF before you click submit

To generate a record that you filled in this form, we recommend to generate a PDF of this page (on a Mac, simply select "print" and then select "print as PDF") before you submit it.

When you submit your (revised) paper to JMIR, please upload the PDF as supplementary file.

Don't worry if some text in the textboxes is cut off, as we still have the complete information in our database. Thank you!

Final step: Click submit !

Click submit so we have your answers in our database!

Submit

Never submit passwords through Google Forms.

Powered by

This content is neither created nor endorsed by Google.

Report Abuse - Terms of Service - Additional Terms
